# The time scales of irreversibility in spontaneous brain activity are altered in obsessive compulsive disorder

**DOI:** 10.3389/fpsyt.2023.1158404

**Published:** 2023-05-10

**Authors:** Davide Bernardi, David Shannahoff-Khalsa, Jeff Sale, Jon A. Wright, Luciano Fadiga, David Papo

**Affiliations:** ^1^Center for Translational Neurophysiology of Speech and Communication, Fondazione Istituto Italiano di Tecnologia, Ferrara, Italy; ^2^BioCircuits Institute, University of California, San Diego, La Jolla, CA, United States; ^3^Center for Integrative Medicine, University of California, San Diego, La Jolla, CA, United States; ^4^The Khalsa Foundation for Medical Science, Del Mar, CA, United States; ^5^San Diego Supercomputer Center, University of California, San Diego, La Jolla, CA, United States; ^6^Department of Neuroscience and Rehabilitation, Section of Physiology, University of Ferrara, Ferrara, Italy

**Keywords:** spontaneous brain activity, permutation entropy, time-reversal symmetry, time scales, dynamical disease, obsessive-compulsive disorder, Kundalini Yoga, meditation

## Abstract

We study how obsessive-compulsive disorder (OCD) affects the complexity and time-reversal symmetry-breaking (irreversibility) of the brain resting-state activity as measured by magnetoencephalography (MEG). Comparing MEG recordings from OCD patients and age/sex matched control subjects, we find that irreversibility is more concentrated at faster time scales and more uniformly distributed across different channels of the same hemisphere in OCD patients than in control subjects. Furthermore, the interhemispheric asymmetry between homologous areas of OCD patients and controls is also markedly different. Some of these differences were reduced by 1-year of Kundalini Yoga meditation treatment. Taken together, these results suggest that OCD alters the dynamic attractor of the brain's resting state and hint at a possible novel neurophysiological characterization of this psychiatric disorder and how this therapy can possibly modulate brain function.

## 1. Introduction

Several important problems in neuroscience boil down to identifying the time scales of neurophysiological associated to cognitive phenomena (1–7). The most obvious example is the determination of a given phenomenon's duration or subdivision into meaningful segments. When the phenomenon at hand has no characteristic duration, as it is the case for spontaneous brain activity or cognitive functions such as thinking or reasoning, identifying time scales involves determining subtler temporal characteristics (8).

There are two main facets to the experimental identification of time scales. The first involves choosing technical apparatus, experimental procedures, and methods of data analysis. Appropriate experiments must ensure that the experimental apparatus can access scales much smaller than the phenomenon's duration time, which, in turn, should be shorter than the observation time. The range of accessible time scales ultimately coincides with that permitted by the methods of data quantification (5). The second part of time scale identification is the choice of variables used to characterize a given system or phenomenon. In neuroimaging or electrophysiological experiments the chosen variable is typically some function of the signal amplitude recorded at a given location. The key point is that each of these aspects comes with its own set of characteristic scales.

Spontaneous brain activity is characterized by nonrandom structure (9, 10), in which patterns are reedited in a nonrandom way across the cortical space (11–14). At various spatial scales, spontaneous brain activity fluctuations display non-trivial statistical and dynamical long-time properties, including scale-freeness (15–19), non-Gaussianity (20), weak-ergodicity breaking (21), and intermittency (19, 22). Hence, spontaneous brain activity has a structure in a wide range of time scales, so that there is not just one but a plurality of time scales, and possibly some relationship among them. These properties, which are mirrored by behavioral fluctuations (23), are altered in various brain pathologies (24, 25), and are modulated by the execution of cognitive tasks (26–31) or by pharmacological manipulations (32). Insofar as these properties appear to be generic, they can be used to characterize both healthy and pathological spontaneous brain activity. Likewise, the effects of experimental variables, including cognitive tasks, neurological or psychiatric pathologies and interventions of various kinds can be quantified in terms of modulations of such properties (8, 33).

Physiological disorders may correspond to changes in the system parameters that lead to bifurcations in the dynamics, which, in turn, correspond to qualitative changes in the observed neural activity or in behavior (34–38). Because brain activity can be understood as the output of underlying nonlinear dynamical processes, nonlinear methods (39–42) are needed to characterize both healthy and pathological brain activity (43–46). One of these methods is the quantification of the temporal irreversibility of the brain activity.

Living systems operate far from equilibrium (47), and this is reflected by the breakdown of time-reversal symmetry. From a statistical viewpoint, time-reversal symmetry quantifies the extent to which it is possible to discern a preferred time direction in the realization of some stationary stochastic process (48). Observed phenomena are thought of as realizations of a stochastic dynamical process, and the goal is to try to extract information on the statistical properties of these processes from the time-reversal symmetry and its breakdown (49–51). For instance, linear Gaussian random processes and static non-linear transformations of such processes are time-reversible, so that time irreversibility implies ruling out Gaussian linear models and their static nonlinear transformations as possible generative models.

Irreversibility can be associated with a characteristic scale, i.e., the scale over which the process manifests time-reversal symmetry breaking (52). In inherently multiscale systems such as living systems, irreversibility may present complex scale-dependence, the breaking of time-reversal symmetry possibly manifesting in different ways at different spatial and temporal scales, thereby inducing a multiplicity of characteristic scales (53, 54). Consistent with the role of nonlinearity and its inherently out-of-equilibrium nature, spontaneous brain activity has long been associated with marked time reversal symmetry breaking (55). The magnitude of irreversibility is characterized by temporal fluctuations and is modulated in a task-specific way (56, 57), with greater values for task-related relative to resting brain activity (57), and wakefulness relative to in deep anesthesia (58). Irreversibility of resting brain activity has also been shown to be altered in various neurological and psychiatric pathologies, including epilepsy (56, 59, 60), attention deficit hyperactivity disorder (57), Alzheimer's disease (56), Parkinson's disease (56), bipolar disorder (57), and schizophrenia (56, 57). These alterations have been shown to be both pathology and frequency-specific (56).

Obsessive-compulsive disorder (OCD) is a psychiatric disorder characterized by anxiogenic, undesired, and recurrent thoughts, experienced as intrusive, distressing, and inappropriate, and by repetitive and time-consuming behaviors or mental acts that the patient is compelled to engage in, often in order to neutralize obsession-induced anxiety (61).

Various strategies for the treatment of patients with OCD have been proposed (62, 63). These include pharmacological (64–67), behavioral (64, 68, 69), and neurostimulation therapies (70, 71). While serotonin re-uptake inhibitors (SRIs) are the only FDA approved drugs, 50% of patients can be considered non-responders when using a 25% to 35% improvement criterion with the Yale-Brown Obsessive-Compulsive Scale (Y-BOCS), and 30% are non-responders to combined first-line therapies (SRIs; exposure and response prevention). Kundalini Yoga meditation was shown to lead to significant improvement in symptoms using the Y-BOCS in patients unresponsive to first line therapies and previously untreated patients (72–77).

Neuroimaging studies have consistently associated OCD's pathophysiology with abnormalities of orbitofronto-striatal structures (78–82), corroborating frontal lobe dysfunction hypothesis (83). However, OCD's electrophysiological characterization appears less univocal (84, 85). Several studies reported abnormalities in event-related brain activity at various latencies (86–88). The power spectrum of spontaneous brain activity in OCD was also found to significantly differ from the one associated with healthy brain activity (89–98), the general finding being an increase in delta and theta band power (2–6 Hz) and a decrease in the alpha band (8–10 Hz) (99). Results on power in the beta range were mixed with both region-specific increases (94) and decreases at rest (100) and during hyperventilation (90) in OCD patients with respect to healthy controls. Reports of frequency characteristics in anterior areas have been inconsistent at all frequency bands. For instance, for frontal alpha band (8–10 Hz), some studies reported increases (101, 102), and other ones decreases in power (91, 92). Likewise, both trait-related right-hemisphere resting frontal hypoactivity, corresponding to higher power in the alpha range (100, 103), and no clear hemispheric differences (91, 92) between the OCD and healthy control participants were reported. These inconsistencies may stem from factors including the heterogeneity of patient population comorbidity for depression or the different frequency band definition in several studies (92). More generally, the power spectrum itself constitutes a pure discriminator of several disorders (autism, addiction, PTSD) and is not a reliable individual outcome predictor (85).

OCD was shown to be associated with decreased global field synchronization of multichannel frontal EEG (104, 105), reduced inter-hemispheric coherence (94, 95), particularly in the alpha band, and altered non-linear interhemispheric coherence at various frequency bands (97). Furthermore, multi-frequency band resting-state functional connectivity analyses highlighted reduced resting long-range functional alpha band connectivity in posterior areas, and condition-dependent increased beta band connectivity (106). Graph-theoretical analysis also showed altered topological structure during rest, in the alpha and beta bands (106, 107), with hypo-clustering in the low-alpha band and hypo/hyper-clustering in low-alpha and high-beta bands, respectively (107).

In an effort to propose more specific quantifiers of resting brain activity in OCD, EEG-based nonlinear complexity quantifiers have also been proposed to constitute a biomarker of OCD, and have been used to discriminate obsessive compulsive disorder (108–112) and to predict treatment resistance (113). Specifically, it was reported that OCD patients were characterized by lower EEG complexity at both prefrontal and right fronto-temporal locations with respect to matched healthy control subjects (110).

Though some information can be deduced from the existing electrophysiological literature, none of the studies explicitly addressed the irreversibility of the brain activity and its timescales in OCD. Here, we quantify the irreversibility of spontaneous magnetoencephalographic brain activity in OCD and its possible modulation by an OCD-specific yogic breathing pattern (72, 73, 75–77). Irreversibility can be quantified in various ways [see (114), for a review]. In this study, we use a quantifier of irreversibility based on ordinal patterns (56, 60, 115–117). Since the typical use of ordinal patterns is the estimation of the so-called permutation entropy (118), which is a well-established measure of time-series complexity, we also test to what extent the permutation entropy can discriminate the two conditions. The main focus of the present study, however, is the irreversibility estimated from the ordinal pattern distribution. In particular, we expected that OCD would significantly affect the characteristic time scales at which irreversibility appears, and that these time scales would be distributed differently in space in OCD patients compared to control subjects. We also conjectured that, if time scales are significantly altered by pathology, an effective therapy should shift their properties toward values observed in the healthy population. We note here that only patients who had chosen not to receive medication participated to this MEG study, thus eliminating one possible confound source. The breathing and meditation therapy was the only treatment received by the patients included into the present analysis (74–76).

## 2. Methods

### 2.1. Participants and the OCD-specific protocol

**Figure 1:**
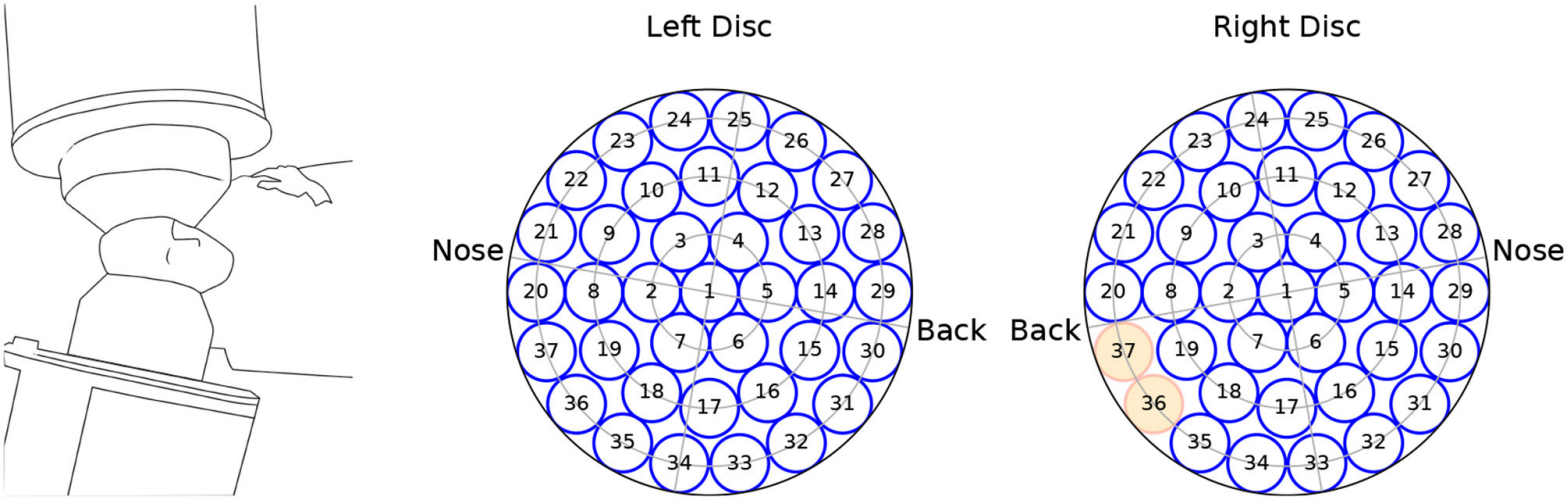
Schematics of the recording equipment **(left)**, and channel arrangement within the **(left, right)** measuring probes. The channels marked in red were not available for the analysis.

We analyzed MEG recordings from 10 OCD patients at baseline (4 men aged 23–36; 6 women aged 25–55) and 9 controls (4 men aged 25–38; 5 women aged 39–64). Controls were matched by sex and roughly by age, except for the two youngest female patients. The MEG recording device allowed performing bilateral measurements on reclining participants. During recording, participants were reclined on their right side as schematically depicted in Figure 1, left.

In a follow-up session 1 year later, all control subjects, otherwise termed healthy controls (HC) were recorded a second time. OCD patients followed a Kundalini-yoga-based breathing therapy over a period of 1 year. The breathing therapy was based on eight primary techniques to be used on a daily basis, and three additional techniques to be used at personal discretion. All techniques are described in detail in (73, 75–77). Three patients did not take the follow-up MEG scan after 1 year. Hence, results for the post therapy condition are based on the remaining 7 patients. After describing the meditation study and the possible adverse effects (i.e., temporary muscle soreness), we obtained written informed consent from all participants, who decided in complete freedom not to receive medication. The study was conducted in compliance with the Code of Ethics of the World Medical Association, Declaration of Helsinki. The analysis of the MEG data continues to receive approval and coverage by the University of California San Diego Human Subjects Internal Review Board.

### 2.2. Data recording and pre-processing

The MEG data were recorded from a dual 37-channel superconducting quantum interference device (Biomagnetic Technology Inc., San Diego, CA) at the Scripps Research Institute (La Jolla, CA). Each channel received the signal from a single detection coil within the biomagnetometer. Detection coils were 20 mm in diameter, and were arranged in concentric circles over a spherically concave surface, which was placed above the auditory cortex and spanned a circular area with a diameter of 144 mm. A scheme of the channels' arrangement is seen in Figure 1. Due to data corruption, two channels from the right hemisphere were not available for analysis.

For each subject, a total recording time of *T*= 1,800 s at a sampling rate of *f*_*s*_ = 231.5 Hz was available. Time series were low-pass filtered by using a forward-backward 8th order Butterworth digital filter with a critical frequency of 45 Hz to ensure that 60 Hz power line artifacts were removed.

### 2.3. Data analysis

#### 2.3.1. Second-order (spectral) statistics

The time series were recorded during a long period of resting-state activity, for which it seems adequate to assume stationarity. We define the autocorrelation function as


(1) $$\begin{array}{l} C_{xx}\left(t\right)=\langle x\left(t^{\prime }\right)x\left(t^{\prime }+t\right)\rangle -{\langle x\rangle }^{2} \end{array}$$

where angular brackets indicate averaging and the stationarity assumption implies that the reference time *t*′ is arbitrary and that the second term does not depend on time. The power spectrum *S*_*xx*_(*f*) is defined as the Fourier transform of *C*_*xx*_(*t*)


(2) $$\begin{array}{l} S_{xx}\left(f\right)=\int dt\;e^{2\pi ift}C_{xx}\left(t\right). \end{array}$$

**Figure 2:**
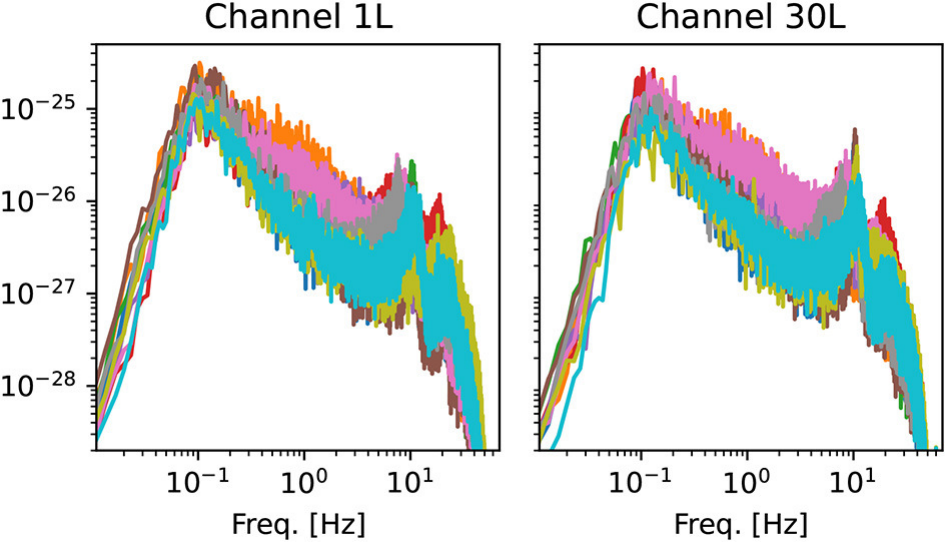
Examples of power spectra from two channels of the left hemisphere. Each line corresponds to one of the 10 OCD patients (baseline condition). Spectra are computed using Welch's method with the same time window used for the estimation of ordinal pattern distributions (see Section 2.3.2).

Some representative power spectra are shown in Figure 2. Although the magnitude and position of spectral peaks varies from patient to patient, the rough shape of the spectra is similar. In particular, there is no sign of divergence of the spectrum in the low-frequency limit *f* → 0. This observation gives some support to the assumption of stationarity (42).

#### 2.3.2. Ordinal patterns

Let *x*_*i*_(*t*) be the time series obtained from channel *i*, where *t* = 1…*N* is the time step (*N* = *T*/Δ*t* = *T*·*f*_*s*_). The time-delay embedding yields a *d* dimensional vector $X_{i}\left(t\right)=\left\{x_{i}\left(t\right),x_{i}\left(t-\bar{\tau }\right),\ldots x_{i}\left(t-\left(d-1\right)\bar{\tau }\right)\right\}$, where τ is the embedding delay measured in time steps and *d* is the embedding dimension (42). In seconds, the embedding delay will be indicated as $\tau =\Delta t\bar{\tau }$. The ordinal pattern π(*X*_*i*_(*t*)) associated to each embedding vector is the permutation of its elements which brings them into ascending order. Since statistics were considered for each channel separately, the channel index will be dropped in the following to reduce notation clutter. The probability of each pattern was estimated as the relative frequency of the pattern within *n*_*w*_ nonoverlapping segments of the total available recording time for each patient. As a matter of fact, Results were qualitatively consistent for values ranging from *n*_*w*_ = 2 to *n*_*w*_ = 10. The standard value of *n*_*w*_ = 6 is used for all figures.

#### 2.3.3. Permutation entropy

Let *p*_*k*_ be the probability of the *k*th permutation pattern. The permutation entropy (PE) is defined as the Shannon entropy of the distribution of ordinal patterns (118)


(3) $$\begin{array}{l} PE_{d}=-\overset{n}{\underset{k=1}{\sum }}p_{k}\log_{2}p_{k}, \end{array}$$

where *n* = *d*! is the number of possible ordinal patterns. The PE is a measure of complexity that has found wide application in the analysis of nonlinear time series (119–121).

#### 2.3.4. Time-symmetry breaking from permutation patterns

Let *p*_*k*_ indicate the probability of the *k*th permutation pattern, as above. We indicate by ${\hat{p}}_{k}$ the ordinal pattern distribution measured from the time-reversed time series, i.e., the probability of observing the *k*th pattern in the time series $\hat{x}\left(t\right)=x\left(N-t\right)$. Studying the time-reversal symmetry breaking has a long tradition and is a hallmark of systems out of equilibrium. It was recently proposed to exploit statistical differences in the ordinal patterns to quantify the irreversibility of time series (60, 116, 122). Here, the measure of time-reversal symmetry breaking is defined as


(4) $$\begin{array}{l} \sigma_{d}\left(\tau \right)=\frac{D\left(p_{k}||{\hat{p}}_{k}\right)}{\tau \left(d-1\right)}=\frac{1}{\tau \left(d-1\right)}\overset{n}{\underset{k=1}{\sum }}p_{k}\log_{2}\frac{p_{k}}{{\hat{p}}_{k}}, \end{array}$$

where $D\left(p_{k}||{\hat{p}}_{k}\right)$ is the Kullback-Leibler (KL) divergence between the ordinal pattern distribution extracted from the original time series and that obtained from the time reversed time-series. Clearly, if the time series is (statistically) symmetric under time-reversal, $p_{k}={\hat{p}}_{k}$ and σ = 0. Any difference in the probability of a pattern and its time-reversed leads to σ>0. Note that although the KL divergence is, in general, not symmetric, in this case the roles of the forward and backward time series can be reversed, which can be seen explicitly as follows. Let ${\overset{⌄}{p}}_{k}$ be the pattern corresponding to the time-reversal of *p*_*k*_. To this end, the pattern labeling can be chosen such that ${\overset{⌄}{p}}_{k}=p_{k+n/2}$ when *k* ≤ *n*/2 and ${\overset{⌄}{p}}_{k}=p_{k-n/2}$ when *k* > *n*/2. Assuming that both *p*_*k*_ and ${\hat{p}}_{k}$ represent the actual probabilities (i.e., that the underlying distribution are perfectly well sampled) implies that ${\overset{⌄}{p}}_{k}={\hat{p}}_{k}$. Then, reversing the roles of *p*_*k*_ and ${\hat{p}}_{k}$ in the definition Equation 4 leads to


(5) $$\begin{array}{l} D\left({\hat{p}}_{k}||p_{k}\right)=\overset{n}{\underset{k=1}{\sum }}{\hat{p}}_{k}\log_{2}\frac{{\hat{p}}_{k}}{p_{k}}\\ \;=\overset{n/2}{\underset{k=1}{\sum }}p_{k+n/2}\log_{2}\frac{p_{k+n/2}}{p_{k}}+\overset{n}{\underset{k=n/2+1}{\sum }}p_{k-n/2}\log_{2}\frac{p_{k-n/2}}{p_{k}}\\ \;=\overset{n}{\underset{k^{\prime }=n/2+1}{\sum }}p_{k^{\prime }}\log_{2}\frac{p_{k^{\prime }}}{p_{k^{\prime }-n/2}}+\overset{n/2}{\underset{k^{\prime }=1}{\sum }}p_{k^{\prime }}\log_{2}\frac{p_{k^{\prime }}}{p_{k^{\prime }+n/2}}=\\ \;=\overset{n}{\underset{k^{\prime }=n/2+1}{\sum }}p_{k^{\prime }}\log_{2}\frac{p_{k^{\prime }}}{{\hat{p}}_{k^{\prime }}}+\overset{n/2}{\underset{k^{\prime }=1}{\sum }}p_{k^{\prime }}\log_{2}\frac{p_{k^{\prime }}}{{\hat{p}}_{k^{\prime }}}\\ \;=\overset{n}{\underset{k^{\prime }=1}{\sum }}p_{k^{\prime }}\log_{2}\frac{p_{k^{\prime }}}{{\hat{p}}_{k^{\prime }}}=D\left(p_{k}||{\hat{p}}_{k}\right), \end{array}$$

which shows that the definition of the IR is symmetric with respect to the choice of forward and backward time series. We will refer to σ_*d*_ as to irreversibility per unit time or, in short, irreversibility rate (IR).

#### 2.3.5. Synthetic data (statistical significance of data)

Because of finite-size effects, it can be ${\overset{⌄}{p}}_{k}\neq {\hat{p}}_{k}$ - and thus σ > 0 - although the underlying system is actually time-reversible. To assess the magnitude of finite-size effects in our data, we generated an ensemble of Gaussian reversible synthetic time-series with the same power-spectrum *S*_*xx*_ as the actual series as described elsewhere (123). Briefly, the series are generated in the frequency domain by drawing two Gaussian random numbers zero mean and variance *S*_*xx*_(*f*)*T*/2 (here *T* = *NΔt*); these two numbers are the real and imaginary part of the Fourier transformed time series, i.e., $ℜ\left[\tilde{x}\left(f\right)\right]$ and $ℑ\left[\tilde{x}\left(f\right)\right]$, respectively. The synthetic time series is then generated via inverse Fourier transform


(6) $$\begin{array}{l} x_{s}\left(t\right)=\int df\;e^{-2\pi ift}\tilde{x}\left(f\right). \end{array}$$

It can be easily seen that the ordinal pattern statistics of these time series are time-reversible:


(7) $$\begin{array}{l} {\hat{x}}_{s}\left(t\right)=x_{s}\left(T-t\right)=\int df\;e^{-2\pi if\left(N-t\right)}\tilde{x}\left(f\right)\\ \;=\int df\;e^{-2\pi ift}{\tilde{x}}^{*}\left(f\right)e^{2\pi iTf}. \end{array}$$

The last equation shows that the time-reversal is equivalent to applying a phase shift to each Fourier component of the original series. Hence, ${\hat{x}}_{s}\left(t\right)$ is a Gaussian stationary series with the same power spectrum as *x*_*s*_(*t*) and it is statistically equivalent to it. Consequently, by applying Equation 4 to the Gaussian noise ensemble, we obtained an empirical distribution of the baseline σ(τ). The significance level was taken as the the 99th percentile of such empirical null distribution.

Another popular, and more refined way, of generating surrogate data is the Iterative Amplitude Adjusted Fourier Transform (124), which attempts to approximately match both the power spectrum and the stationary distribution of the original time series. However, this is an iterative procedure which does not converge to the exact power spectrum and that can possibly introduce unwanted nonlinear structure into the surrogate data. In the present case, we preferred the simpler approach of a Gaussian stationary ensemble with matched power spectrum since it is guaranteed to be time-reversible and that the stationary distribution of the data is not too far from a Gaussian distribution.

#### 2.3.6. Forbidden patterns

A further practical issue is represented by the case when the data yield *p*_*k*_ = 0 for some *k*. In this case, Equation 4 would diverge. To deal with this case, we proceeded as follows. Suppose one pattern is not observed in the dataset at hand. Without loss of generality, we can label patterns such that the unobserved pattern is *p*_1_, i.e., *p*_1_ = 0. We now assume that the actual probability is $p_{1,true}=\epsilon \ll N^{-1}$, where *N* is the total number of samples. In other words, we assume that the missing pattern has a very small non-zero probability, and it is therefore not observed in the data as a result of insufficient statistics. Furthermore, we assume that ${\hat{p}}_{1}\gg N^{-1}$, i.e., the reversed pattern is well estimated by the available data (and, hence, $\hat{p}\gg \epsilon$). Consider the quantity $x\log_{2}\left(x/{\hat{p}}_{1}^{\prime }\right)+{\hat{p}}_{1}^{\prime }\log_{2}\left({\hat{p}}_{1}^{\prime }/x\right)$; its derivative with respect to *x*, $\log_{2}\left(x/{\hat{p}}_{1}^{\prime }\right)+1-{\hat{p}}_{1}^{\prime }/x$ is always negative if $x<{\hat{p}}_{1}^{\prime }$, i.e., it is a decreasing function of *x*. In the same range, it is an increasing function of $p_{1}^{\prime }$. Hence, if we set $p_{1}^{\prime }=N^{-1}$ and rescale all other probabilities as $p_{k}^{\prime }=p_{k}N/\left(N+1\right)$ to preserve normalization, we obtain an irreversibility estimate $\sigma_{d}^{\prime }$ which is, under the above assumptions, a lower bound on the actual irreversibility:


(8) $$\begin{array}{l} \sigma^{\prime }\left(\tau \right)\left(d-1\right)\tau =p_{1}^{\prime }\log_{2}\frac{p_{1}^{\prime }}{{\hat{p}}_{1}^{\prime }}+{\hat{p}}_{1}^{\prime }\log_{2}\frac{{\hat{p}}_{1}^{\prime }}{p_{1}^{\prime }}+\overset{n}{\underset{\begin{array}{c} k=2\\ k\neq 1+n/2 \end{array}}{\sum }}p_{k}^{\prime }\log_{2}\frac{p_{k}^{\prime }}{{\hat{p}}_{k}^{\prime }}\\ \;≲p_{1}^{\prime }\log_{2}\frac{p_{1}^{\prime }}{{\hat{p}}_{1}^{\prime }}+{\hat{p}}_{1}^{\prime }\log_{2}\frac{{\hat{p}}_{1}^{\prime }}{p_{1}^{\prime }}+\underset{\begin{array}{c} k=2\\ k\neq 1+n/2 \end{array}}{\sum }p_{k}\log_{2}\frac{p_{k}}{{\hat{p}}_{k}}\\ \;=N^{-1}\log_{2}\frac{N^{-1}}{{\hat{p}}_{1}^{\prime }}+{\hat{p}}_{1}^{\prime }\log_{2}\frac{{\hat{p}}_{1}^{\prime }}{N^{-1}}+\overset{n}{\underset{\begin{array}{c} k=2\\ k\neq 1+n/2 \end{array}}{\sum }}p_{k}\log_{2}\frac{p_{k}}{{\hat{p}}_{k}}\\ \;<\epsilon \log_{2}\frac{\epsilon }{{\hat{p}}_{1}}+{\hat{p}}_{1}\log_{2}\frac{{\hat{p}}_{1}}{\epsilon }+\overset{n}{\underset{\begin{array}{c} k=2\\ k\neq 1+n/2 \end{array}}{\sum }}p_{k}\log_{2}\frac{p_{k}}{{\hat{p}}_{k}}\\ \;=\sigma \left(\tau \right)\left(d-1\right)\tau . \end{array}$$

Clearly, the above bound is meaningful only if the forbidden patterns are few and the corresponding time-reversed patterns are sufficiently sampled. In the following, we consider *d* = 3, 4, which ensured that both conditions were met. On a final technical note, the usual empirical prescription for the number of samples to be larger than (*d* + 1)! to obtain a good estimate of the PE seems insufficient for a good estimate of the KL divergence, since the ratio of probabilities makes this measure much more sensitive to finite-size errors than the PE.

#### 2.3.7. Statistical tests

For some quantities, a linear mixed model (LMM) was used to asses whether the observed differences are statistically significant. This approach permits to deal with multiple measurements from the same subjects. The model included the subject as a random effect. There were three predictors: (i) belonging to either group, (ii) the 1-year interval (only for the control group), (iii) the 1-year treatment period (only for OCD patients).

## 3. Results

This section is organized as follows. We begin by reporting the PE as a function of the embedding delay in the different experimental conditions. We then measure the IR in the single recording channels. Motivated by the behavior of the IR as a function of the embedding delay, we then define a IR relative to “fast” and “slow” timescales and study how the IR is distributed across channels. Finally, we investigate the interhemispheric symmetry of the IR, i.e., between homologous areas of the two hemispheres.

### 3.1. PE is weakly modulated by OCD

**Figure 3:**
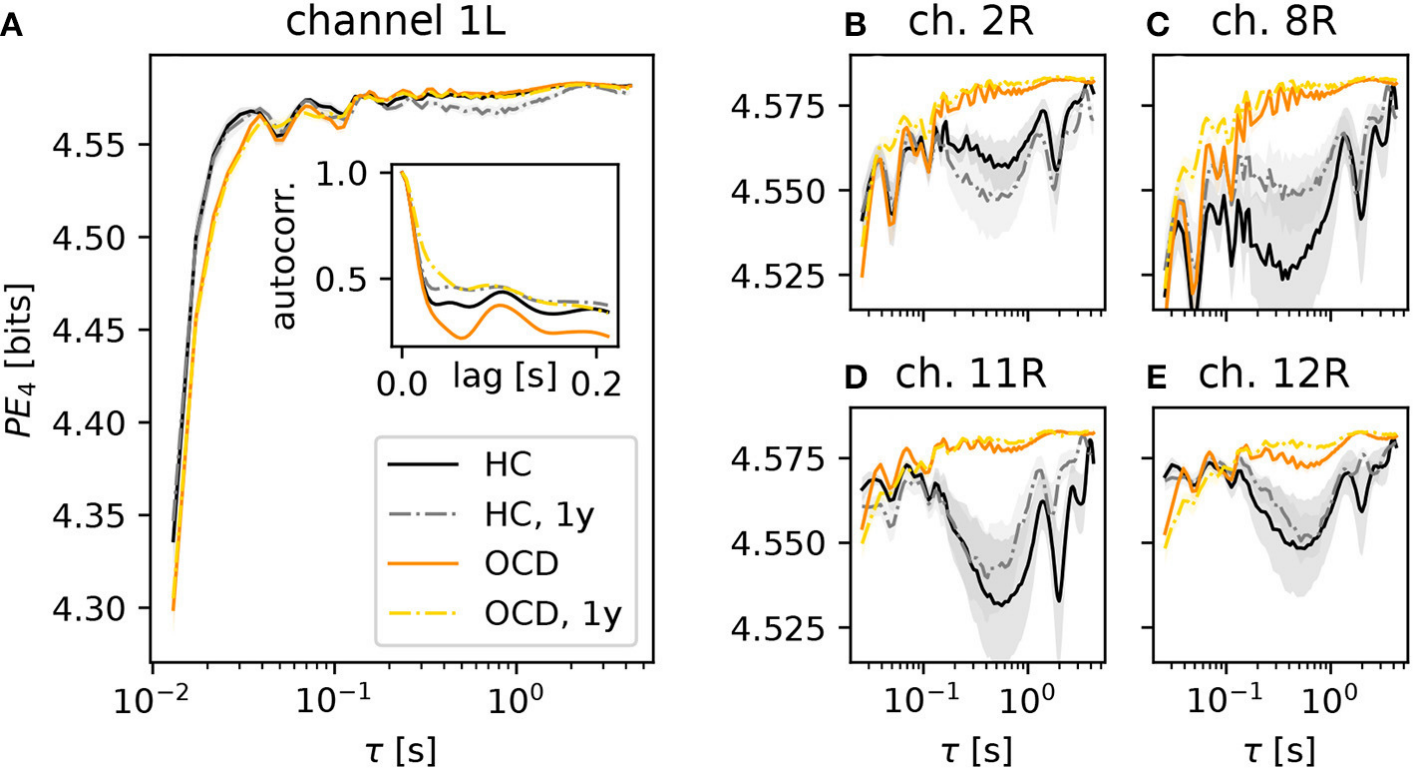
Average permutation entropy (PE) of several representative channels as a function of the embedding delay τ for the different groups and conditions: HC (solid black line: baseline, dashed-dotted gray line: second session 1 year later), OCD patients (orange solid line: baseline, yellow dashed-dotted line: after 1 year of treatment). The embedding dimension is *d* = 4. Panel **(A)** shows the PE measured in the channel 1L. The inset represents the autocorrelation function of the same channel (same color and line coding). Nearly all channels displayed the same qualitative behavior at short τ and the difference between the two groups. Panels **(B–E)**: PE measured in channels 2, 8, 11, and 12 of the right disc as indicated above each panel, respectively (same color and line style coding). Results from channel 25R are also qualitatively similar to channels 2, 8, 11 and 12 (not shown). Shaded areas represent one-SD confidence intervals for the mean over subjects and time windows.

We begin by considering the PE as a function of the embedding delay τ, averaged across subjects in a specific group and condition. For brevity, we focus on the case *d* = 4 (results for *d* = 3 are qualitatively similar). Figure 3 shows results for several representative channels. The PE in channel 1 of the left hemisphere (Figure 3A) increased on average more rapidly (as a function of the embedding delay) in the control subjects (black solid line) than in OCD patients (orange solid line). This difference persisted in the second recording for both groups (gray dashed-dotted line: control; yellow dashed-dotted line: patients), the results of which are consistent with the first recording. The likely reason for this different behavior of the PE in the two groups is the different time-course of the autocorrelation function of the two groups (Figure 3A, inset). The first minimum of the autocorrelation function occurred at shorter time lags for control subjects than for OCD patients. The time lag corresponding to the minimum roughly corresponds to the τ at which the PE reaches saturation, which is in line with what is expected from the theory of time-delay embedding (39). The faster increase of the PE as a function of τ in the control group was consistently observed in nearly all channels (not shown).

A further difference between the two groups was observed in several channels of the right hemisphere, shown in the four panels in the right side of Figure 3 (color and line coding as in Figure 3A). The PE in channel 2R (Figure 3B) displays a difference in the two groups: the PE of control subjects is lower than that of OCD patients over a wide range of embedding delays (from about 0.2 s to ~3 s). This difference is not explainable in terms of the (linear) autocorrelation in a straightforward manner. The PE as a function of τ was rather consistent in the two recording sessions for the control group. It is noteworthy that the shape of the PE curve of OCD patients was hardly affected by treatment, as shown by the strong similarity of orange solid line and yellow dashed-dotted lines in Figure 3B. Figure 3C shows the same qualitative behavior of the PE as a function of the embedding delay τ in channel 8R, which is next to channel 2R. Here, the difference between the two groups is more pronounced than in channel 2R. A second group of channels on the upper front side of the right hemisphere displays the same qualitative picture, as it can be seen in Figure 3D (channel 11R) and Figure 3E (results from channel 12R). Similar observations can be made in channel 25R, which is next to channel 11R (not shown).

### 3.2. Different modulation of IR at fast and slow time scales

**Figure 4:**
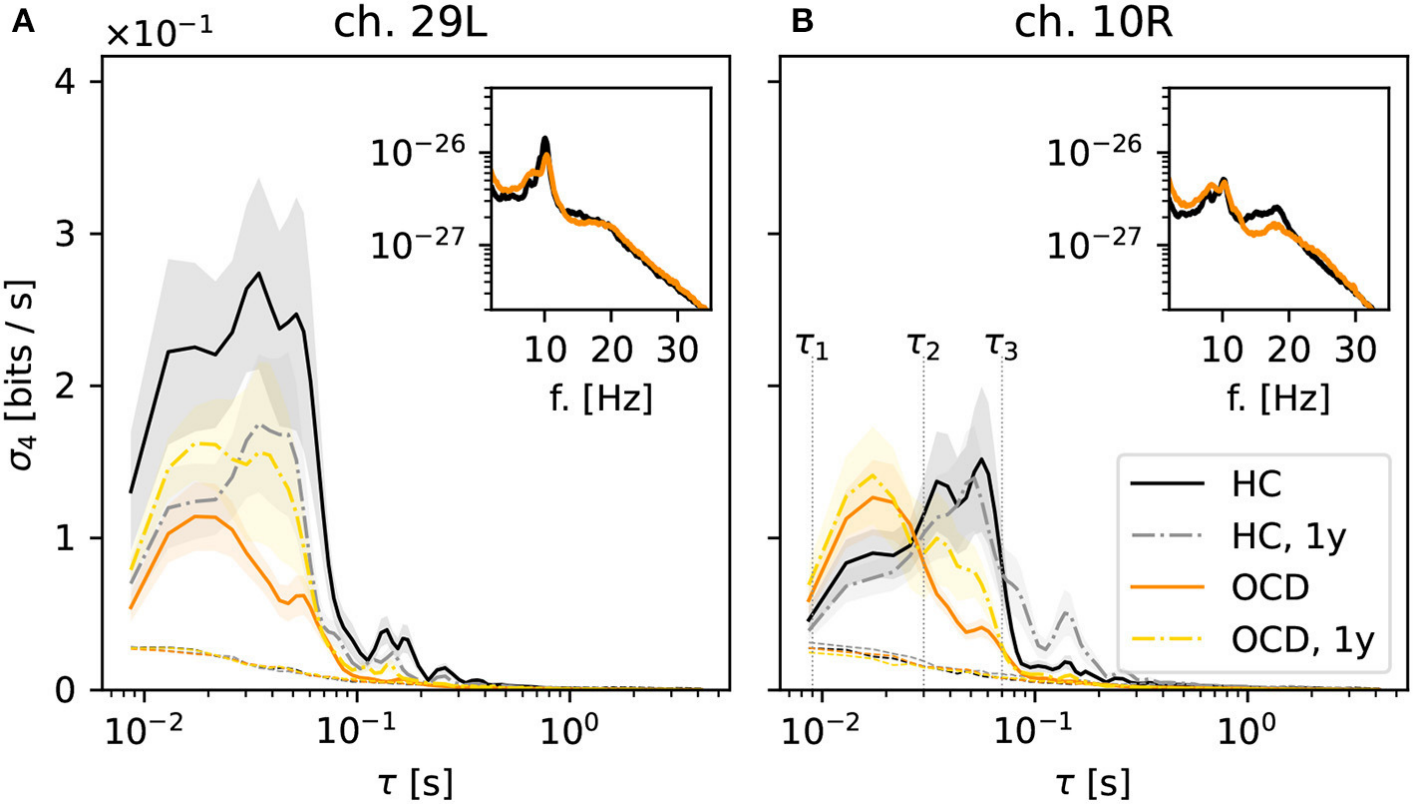
Average irreversibility rate (IR) of two representative channels [29L in panel **(A)** and 10R in panel **(B)**] as a function of the embedding delay τ for the different groups and conditions: controls (HC: solid black line: baseline, dashed-dotted gray line: second session 1 year later), patients (OCD: orange solid line: baseline, yellow dashed-dotted line: after 1 year of treatment). Insets show the average power spectrum of the respective channels in baseline condition. In OCD patients the IR mostly peaks at faster time scales (τ ~ 15ms), whereas in HC the IR can peak at slower time scales (τ ~ 50ms).

Next, we turn to the behavior of the irreversibility rate (IR) σ_*d*_(τ) as a function of the embedding delay τ, again focusing on the case *d* = 4. The qualitative behavior of σ_4_(τ) observed in most channels of the control group recordings can be roughly categorized in two cases, which are illustrated by the two representative channels shown in Figure 4. In some cases, represented by channel 29L, a significant IR is observed for embedding delays up to ≈60 ms (Figure 4A, black solid line), where it sharply drops to levels just close to the threshold for statistical significance (black dotted lines). Some mild IR can still be measured in the range 0.1 s to 0.3 s, above which no significant difference from the null level can be observed. The qualitative shape of σ_4_ as a function of τ in the follow-up recording session 1 year later (gray dashed-dotted line) is consistent with the first session, although the overall magnitude is roughly halved. In other cases, as for channel 10R (Figure 4B), σ_4_ measured in control subjects (black solid line) displays a pronounced peak in the range 30 ms to 60 ms, above which the same sharp drop is seen as in the other channel. In this case, the results of the second recording session 1 year later (gray dashed-dotted line) are both qualitatively and quantitatively consistent with the first session.

In OCD patients, the shape of the IR curve is markedly different. At baseline, it attains a pronounced peak between 10 and 20 ms, above which it strongly declines to the significance threshold. Above τ ≈ 70 ms no significant IR is observed. This behavior is seen rather consistently in both channels displayed in Figure 4.

Remarkably, the qualitative shape of σ_4_(τ) in patients after 1-year treatment (Figure 4, yellow dashed-dotted line) is in-between that of patients and controls at baseline: Similar to baseline condition, a peak around 15 ms is observed. However, σ_4_ decreases more slowly on increasing τ compared to the untreated case. A small secondary peak is seen for τ ≈ 30 ms, i.e., where σ_4_(τ) is maximum for control subjects. This shape of the curve is similar in both considered channels.

Overall, the results of Figure 4 suggest that the timescales of the IR estimated from permutation patterns differ in the two groups. To investigate whether these results can be easily explained in terms of differences in the power spectra, the insets in Figure 4 show the average power spectra of respective channels. More specifically, the inset in Figure 4A shows the average power spectrum of channel 29L in baseline conditions for HC (black line) and OCD patients (orange line). The two spectra are quite similar in shape and magnitude over the entire frequency axis. The most prominent oscillation is seen around 9 Hz. The power spectra measured from channel 10R, shown in the inset of Figure 4B are similar to the previous case, except that the peak around 9 Hz is less marked for both groups, and that in the range 10 Hz to 20 Hz OCD patients have slightly less power. Although there is no straightforward link between the peaks in the power spectrum and the time scales of the IR, there seem to be no clear-cut discrepancy in the power spectra of the two groups that could clearly explain the different timescales emerging from the data of Figure 4.

Going back to Figure 4B, the behavior of σ_4_(τ) suggests the definition of two ranges of time scales, the edges of which are marked by vertical dotted lines in Figure 4B: the “fast” time range Δτ_*f*_ which goes from τ_1_ = 8 ms to τ_2_ = 30 ms and the “slow” time range Δτ_*s*_ which spans from τ_2_ = 30 ms to τ_3_ = 70 ms. Looking at the results of Figure 4, the largest share of the IR in OCD patients is concentrated in the “fast” range (τ_1_, τ_2_), whereas in the control HC group the IR is either stronger in the “slow” range (τ_2_, τ_3_), as in channel 10R or more evenly distributed, as in channel 29L.

**Figure 5:**
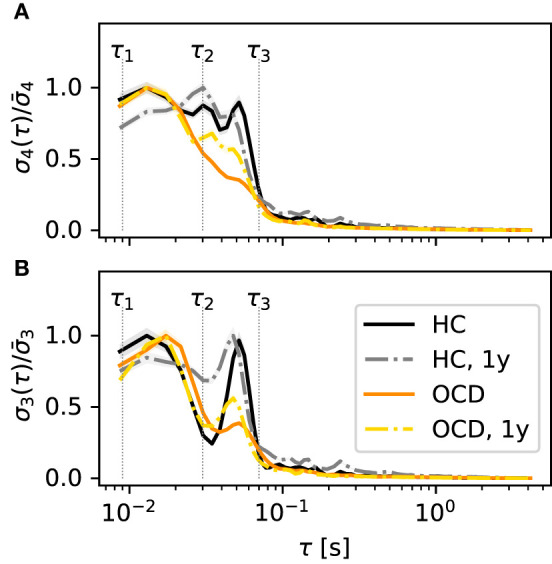
Time scales of the IR are qualitatively different in the various conditions and for the two embedding dimensions *d* = 4 **(A)** and *d* = 3 **(B)**. Normalized IR rate $\sigma \left(\tau \right)/\bar{\sigma }$ averaged over all channels of the left disc as a function of the embedding delay τ. Color and line coding: healthy controls (HC; black solid: baseline; gray dashed dotted: follow-up 1y later), OCD patients (orange solid line: baseline, yellow dashed-dotted: follow-up 1y later).

A difference in “fast” and “slow” IR persists also if all channels are considered altogether. Figure 5A shows $\sigma_{4}\left(\tau \right)/{\bar{\sigma }}_{4}$, the IR averaged over all channels in the left hemisphere, normalized to the maximum of each curve $\bar{\sigma_{4}}$. The color coding is as in the previous figures. It can be seen that $\sigma_{4}\left(\tau \right)/{\bar{\sigma }}_{4}$ stays close to its maximum value in the entire range 10 ms to 70 ms, which spans both “fast” and “slow” ranges as defined above, when control subjects are considered (black solid line). This finding is confirmed by the second recording session, in which $\sigma_{4}\left(\tau \right)/{\bar{\sigma }}_{4}$ is above 80% of its maximum value over the same range (gray dashed-dotted line). In contrast, $\sigma_{4}\left(\tau \right)/\bar{\sigma_{4}}$ peaks around ≈15 ms for OCD patients and then drops faster to zero (orange solid line). One year after treatment the decrease is slower, although the peak is still clearly visible at the same time scale as at baseline.

A relevant issue is whether the picture changes if a different embedding dimension *d* is used. In principle, increasing *d* would allow for a finer probe of the state space. However, for *d* = 5, the number of unobserved patterns is too large to obtain a reliable estimate of σ_5_. Hence, we will compare the results discussed so far to the case *d* = 3. For brevity, we will focus on the averaged normalized IR $\sigma_{3}/{\bar{\sigma }}_{3}$, which is shown in Figure 5B (color and line coding as in Figure 5A). The general picture bears several resemblances with the case *d* = 4: in control subjects, the IR is large in the middle of both “fast” and “slow” ranges large with two peaks at ≈20 ms and ≈60 ms, respectively. Above this value of τ, $\sigma_{3}/{\bar{\sigma }}_{3}$ rapidly drops. Furthermore, $\sigma_{3}/{\bar{\sigma }}_{3}$ measured in OCD patients peaks around 20 ms, above which it rapidly drops. There is, however, also the following difference to the case *d* = 4: In both groups, $\sigma_{3}/{\bar{\sigma }}_{3}$ shows a dip around τ_2_, which was not evident in σ_4_. This dip is more pronounced in control subjects. Despite this difference, Figure 5 shows an overall picture that is roughly consistent for the two embedding dimensions *d* = 3, 4: If all channels are lumped together, the IR is more concentrated in the “fast” range in OCD patients, whereas it is more evenly distributed when the control group is considered.

### 3.3. Contrasting spatial distribution of fast and slow IR

In the following, we will investigate whether the difference between the two groups observed in Figures 4, 5 is spatially structured over the two hemispheres in some way. To this end, we define the average IR in the “fast” and “slow” ranges for each channel as


(9) $$\begin{array}{l} \omega_{f}\left(i\right)=\frac{1}{\tau_{2}-\tau_{1}}\int_{\tau_{1}}^{\tau_{2}}d\tau \;\sigma_{4,i}\left(\tau \right),\;\omega_{s}\left(i\right)=\frac{1}{\tau_{3}-\tau_{2}}\int_{\tau_{2}}^{\tau_{3}}d\tau \;\sigma_{4,i}\left(\tau \right), \end{array}$$

respectively. In Equation 9, σ_4, *i*_(τ) is the IR rate estimated for channel *i* from ordinal patterns with embedding dimension *d* = 4, as defined in Equation 4, and the two integration ranges are defined above and indicated in Figures 4, 5.

**Figure 6:**
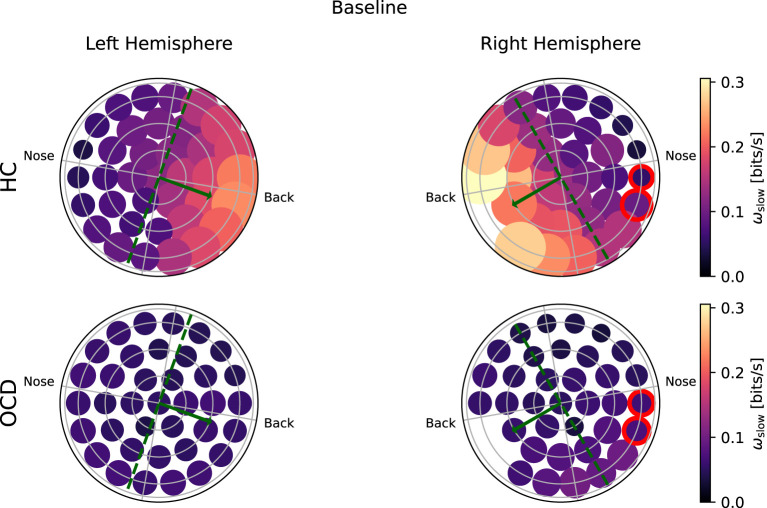
Topological representation of irreversibility rate at slower time scales (ω_*s*_) in all channels at baseline. **(Top row)**: Controls (HC); **(bottom row)**: OCD patients. Note that ω_*s*_ is asymmetrically distributed in HC and more uniform in OCD patients.

**Figure 7:**
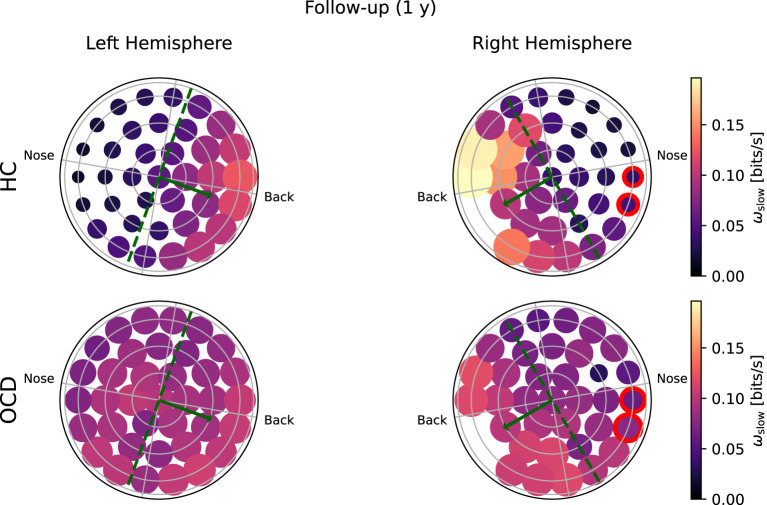
Topological representation of average irreversibility rate at slow time scales (ω_*s*_) in all channels in follow-up recording 1 year after the first recording. **(Top row)**: Controls (HC); **(bottom row)**: OCD patients. Compared to baseline, the overall values ω_*s*_ are smaller in HC than at baseline, but the asymmetrical distribution persists. In OCD patients, ω_*s*_ is generally larger than at baseline. It is still rather uniformly distributed, although in the right hemisphere there seem to be a mild increase in the asymmetry with respect to the top-bottom axis.

Figure 6 shows how the average IR rate in the “slow” range ω_*s*_(*i*) is distributed over the two hemispheres at baseline. In the HC group (top row) the spatial distribution of ω_*s*_(*i*) exhibits a clear asymmetry with respect to the top-bottom axis, which is roughly aligned with the intersection of a coronal plane with the center of the recording probe (green dashed line in Figures 6, 7): In both hemispheres, ω_*s*_(*i*) is larger in the back side, especially toward the back edge. In OCD patients (Figure 6, bottom row) the picture is markedly different. The magnitude of ω_*s*_(*i*) is generally lower; the most striking difference, however, is its rather uniform spatial distribution in both hemispheres.

In the follow-up recording taken 1 year later, the absolute values of ω_*s*_(*i*) measured in the HC group are generally somewhat smaller in magnitude (Figure 7, top line). However, the asymmetric distribution of ω_*s*_(*i*) is consistently observed, with a higher concentration of “slow” IR in the back side of both hemispheres. Results for OCD patients (Figure 7, bottom row) show that ω_*s*_(*i*) is generally larger in magnitude than at baseline. The spatial distribution is still rather uniform in the left hemisphere. In the right hemisphere (Figure 7, bottom right), some mild asymmetry with respect to the top-bottom axis is present, with channels located at the back side of the probe measuring a slightly higher ω_*s*_ than those on the other side. In this sense, there has been a change in OCD patients that makes the spatial distribution of ω_*s*_ more similar to that of control subjects, although the magnitude of this change is moderate.

**Figure 8:**
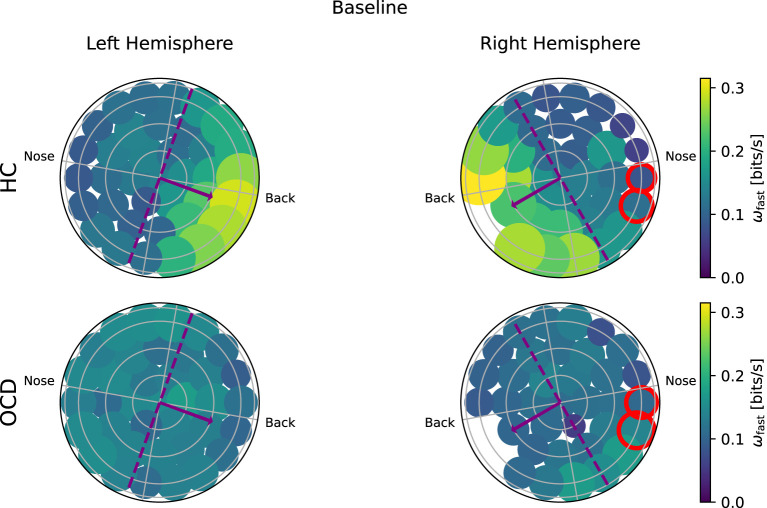
Topological representation of IR at faster time scales (ω_*f*_) in all channels at baseline. **(Top row)**: Controls (HC); **(bottom row)**: OCD patients. There is a strong asymmetry in how (ω_*f*_) is spatially distributed when control subjects (HC) are considered, with the larger part of the fast IR concentrated toward the back area. In OCD patients, (ω_*f*_) is more homogeneously distributed.

Turning to the “fast” IR ω_*f*_(*i*), Figure 8 shows that in the HC group (top row) a clear asymmetry in the distribution exists, which looks similar to that observed for the “slow” IR: a larger ω_*f*_ is measured from channels located toward the back. The overall magnitude of ω_*f*_ measured in OCD patients (Figure 8, bottom row) is similar to that of HC. However, the spatial distribution is rather uniform, in both hemispheres.

**Figure 9:**
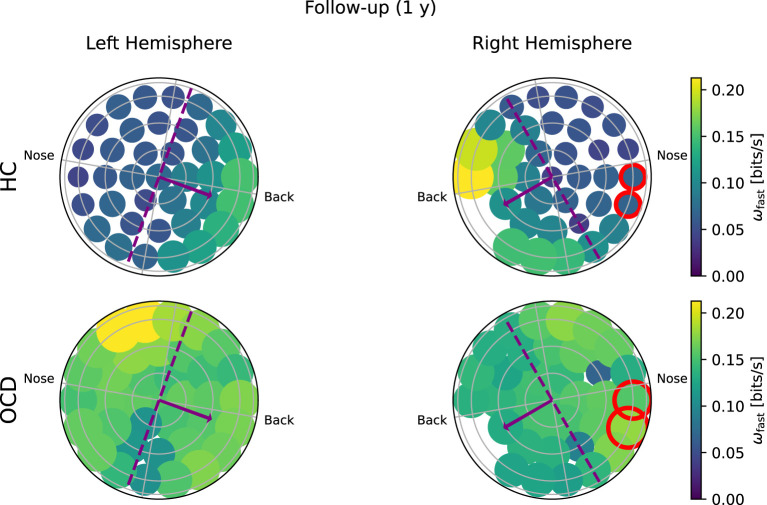
Topological representation of IR at faster time scales (ω_*f*_) in all channels in the follow up session. **(Top row)**: Controls (HC); **(bottom row)**: OCD patients. The overall IR is reduced, but the spatial distribution of ω_*f*_ is still rather asymmetrically concentrated toward the back in HC and more uniform in OCD patients.

In the follow-up recording, the magnitude ω_*f*_ in the HC group (Figure 9, top row) is generally somewhat smaller than in the first recording, but the asymmetric spatial arrangement is clearly conserved. In OCD patients, there are no major changes in the overall magnitude of ω_*f*_. The spatial distribution is still rather uniform, although some mild asymmetry can be observed in the opposite direction, i.e., toward the front side.

**Figure 10:**
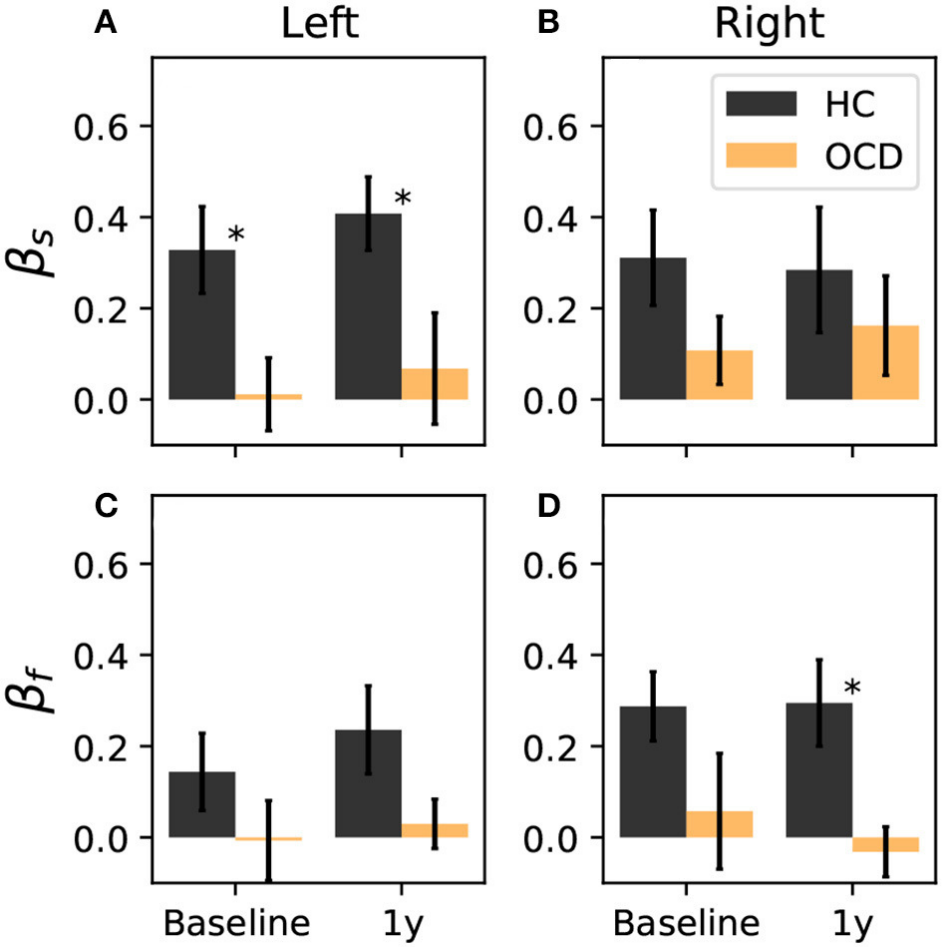
Coronal-plane intrahemispheric asymmetry of IR at slow **(A, B)** and fast time scales **(C, D)** in the left and right hemispheres. Asymmetry indexes are defined in Equation 10. Black bars: controls; orange bars: OCD patients. Results from the LMM regression indicate that the only statistically significant predictor is belonging to either groups (*p* = 0.007), when β_slow_ in the left hemisphere is considered, which is marked by asterisks.

The overall picture emerging from Figures 6–9 suggests that the asymmetric distribution of ω_*f*_ and ω_*s*_ with respect is a trait that is consistently characterizing the control group with respect to OCD patients, rather than the overall magnitude of the same quantities. To make this observation quantitative, we define a intrahemispheric asymmetry index for the two quantities as follows:


(10) $$\begin{array}{l} \beta slow=\frac{\sum_{i}\omega_{slow}\left(i\right)\rho \left(i\right)}{\sum_{i}\omega_{slow}\left(i\right)|\rho \left(i\right)|};\;\beta_{fast}=\frac{\sum_{i}\omega_{fast}\left(i\right)\rho \left(i\right)}{\sum_{i}\omega_{fast}\left(i\right)|\rho \left(i\right)|}, \end{array}$$

where ρ(*i*) is the signed distance of channel *i* to the symmetry axis, where the axis is indicated as a green dashed line in Figures 6, 7 and as a purple dashed line in Figures 8, 9. The positive direction is toward the back side, as indicated by the arrows in Figures 6–9. To ensure that β_*s*_ and β_*f*_ are equal to zero if the distribution is uniform, the two channels that are located at symmetric positions of the two unavailable channels in the right hemisphere are discarded from the calculation of β_*s*_ and β_*f*_. These two channels are circled in red in Figures 6–9. Furthermore, both asymmetry indexes are defined such that −1 < β_*s*_ < 1 and −1 < β_*f*_ < 1, where a positive β_*f*_ (β_*s*_) indicates that ω_*f*_ (ω_*s*_) is more concentrated toward the back, whereas a negative value of the asymmetry indicates that the spatial distribution has more weight toward the front side. Figure 10A reports the asymmetry index of the “slow” IR ω_*s*_ in the left hemisphere. Here, the asymmetry is significantly larger for the control subjects than for OCD patients both at baseline and follow-up conditions, which yield similar values of β_*s*_. Indeed, the LMM regression yields the group as the only statistically significant predictor (*p* = 0.007). In the right hemisphere (Figure 10B), the average asymmetry index toward the back side is significantly large than zero in HC (*p* = 0.04, t-test) whereas it very close to zero for OCD patients (*p* = 0.97, t-test). However, the difference between the two groups is not large enough to be a significant predictor. After 1y, the HC group displays similar average values of β_*s*_; the asymmetry index of OCD grows slightly, but not enough to be considered significantly different from zero. The asymmetry index of “fast” IR in the left hemisphere is reported in Figure 10C. The mean β_*f*_ is larger than zero for HC in both recording sessions by more than one standard deviation while in OCD patients, the mean β_*f*_ is close to zero. However, the difference between the two groups is not large enough to be considered statistically significant with the data at hand, and the LMM does not yield any statistically significant predictor (the strongest effect is the group, for which *p* = 0.09). Finally, β_*f*_ in the right hemisphere at baseline is similar to that measured in the left hemisphere in both groups, with HC displaying some modest asymmetry and OCD patients no coronal-plane asymmetry. In the follow-up recording, the mean β_*f*_ is unchanged in both groups, and the LMM analysis yields no statistically significant predictor for this case.

Overall, the intrahemispheric asymmetry of the “slow” IR in the left hemisphere is the strongest difference as far as the spatial distributions are concerned, in that this asymmetry is essentially absent in OCD patients, whereas it is consistently observed in control subjects. In the other hemisphere and for the “fast” IR, the same tendency can be observed, but more data are needed to give stronger support to this observation.

### 3.4. OCD modulates the interhemispheric asymmetry of IR

**Figure 11:**
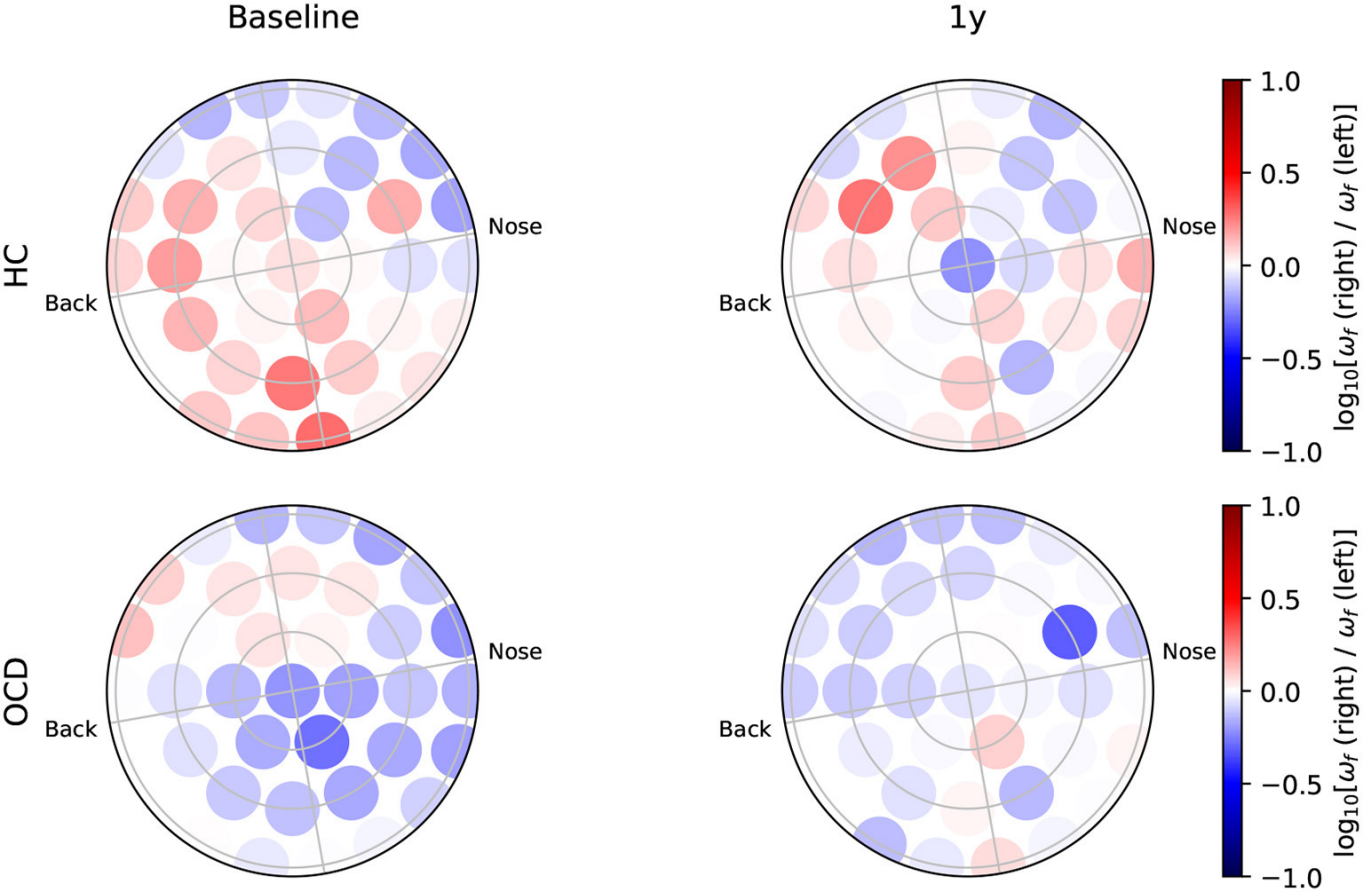
Topological representation of IR **(right, left)** asymmetry for slow IR.

Finally, we investigate how different homologous brain regions are with respect to the IR, i.e., the interhemispheric (a) symmetry of the IR. To this end, we can compare the log-ratio of ω_*s*_ and ω_*f*_ for corresponding channels in the two hemispheres. More precisely, we consider the ratio log_10_[ω_*s*_(*i*)/ω_*s*_(α(*i*))] where *i* is the *i*th channel of the right side, and α(*i*) is the corresponding channel of the left side with respect to the left/right symmetry axis. This interhemispheric asymmetry log-ratio is shown in Figure 11 with a diverging color code (see the color bar), so that dark red circles indicates strong asymmetry toward the right side, dark blue circles mark strong asymmetry toward the left hemisphere, and white circles depict channels for which the average ω_*s*_ is symmetric in the two hemispheres. At baseline (**Figure 13**, top left), control subjects show a clear asymmetry toward the right side in most channels. In the follow-up session (**Figure 13**, top right), the average ratio log_10_[ω_*s*_(*i*)/ω_*s*_(α(*i*))] is positive in the majority of channels, although there is a larger number of channels in which the ratio is close to zero or mildly negative. In OCD patients (Figure 11, bottom left), there are many channels for which the asymmetry log ratio is very close to zero, and an equally number of moderately positive (mostly in the back side) and negative values (mostly in the front side). In the follow-up session (Figure 11, bottom right), there are no major changes with respect to the first recording.

**Figure 12:**
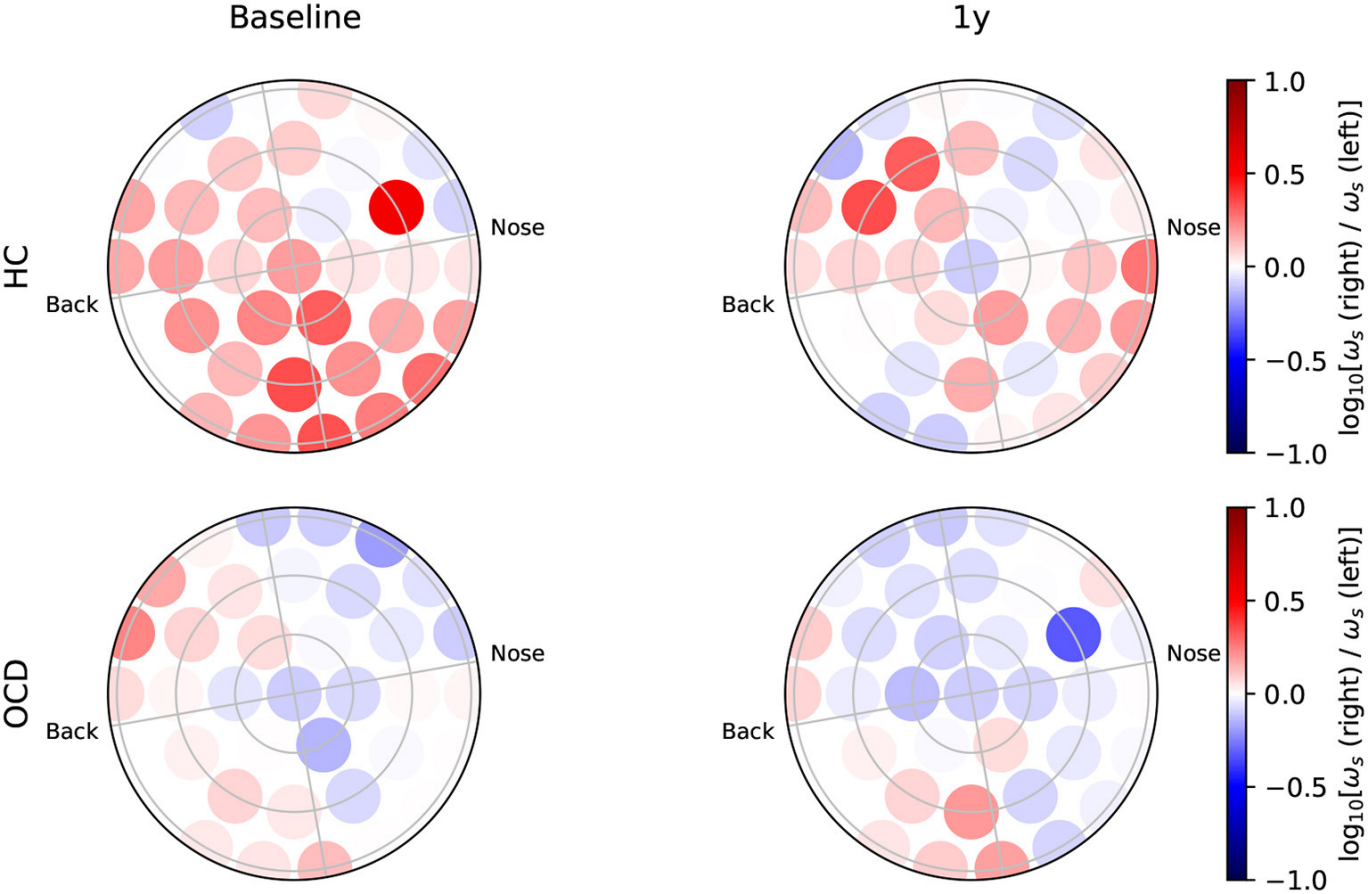
Topological representation of IR **(right, left)** asymmetry for fast IR.

Figure 12, right, left asymmetry ratio of the “fast” IR, log_10_[ω_*f*_(*i*)/ω_*f*_(α(*i*))]. Results for control subjects during the first session (Figure 12, top right) indicate that the “fast” IR is stronger in the right side for channels located at the back, and stronger in the left side for channels located toward the top and front areas. In the follow-up recording, the spatial distribution is roughly similar, although there are several more channels in which the log ratio is very close to zero, which indicates equally strong fast IR in the two homologous areas. In OCD patients the picture is markedly different: in Figure 12, bottom left, most channels are marked in blue, which indicates that the “fast” IR is larger in the left hemisphere. In the follow-up recording (Figure 12, bottom left), the ratio is weaker and close to zero in several channels, but in most cases ω_*f*_ is stronger in the left side.

**Figure 13:**
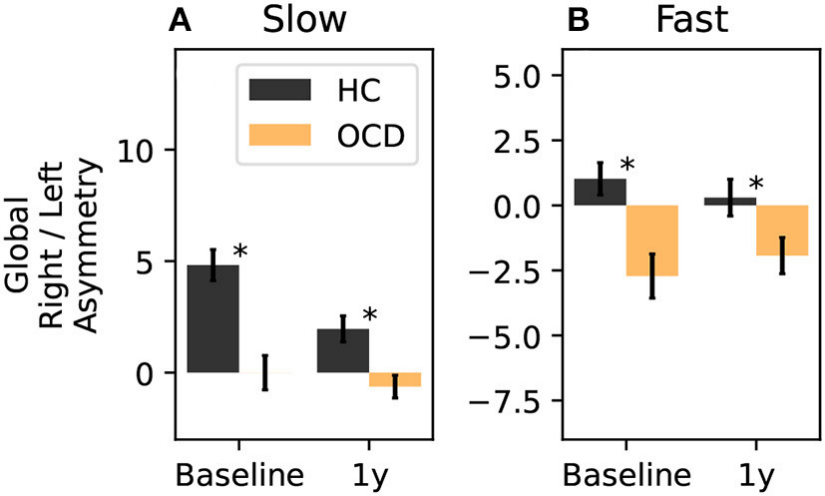
Total right-left asymmetry for slow **(A)** and fast **(B)** IR. The LMM regression indicates only belonging to one group as statistically significant predictor (*p* = 0.008 for the slow IR, and *p* = 0.038 for the fast IR). The asterisk marks a statistically significant difference between groups.

Finally, we can consider a global asymmetry ratio as the sum over all channels of the log-ratio considered so far. Figure 13A shows the global asymmetry of the slow IR, which is clearly positive (i.e., stronger in the right side) in control subjects, and zero in OCD patients. The LMM regression yields a statistically significant effect for the group fixed effect (*p* = 0.008), and no significant effect for the other two predictors. The fast IR behaves differently (Figure 13B): it is very close to being symmetric in control subjects, and it is negative (i.e., predominant in the left) in OCD patients. The LMM analysis yields again the group as the only statistically significant effect (*p* = 0.038).

## 4. Discussion

In the present study, we sought signatures of OCD in multichannel MEG recordings obtained at resting state over an extended period of time. The general framework of our analysis was that of the symbolic dynamics defined by ordinal patterns. Typically, ordinal patterns are used to compute the PE (119), which is a measure of complexity. How OCD affects the complexity of brain activity patterns was the topic of previous studies (108–112). Here the main tool to characterize brain activity and its time scales was instead a measure of time-symmetry breaking, which we called irreversibility rate (IR). The IR was defined as the Kullback-Leibler (KL) divergence between the ordinal pattern distribution observed in the original time series and that measured from the time-reversed counterparts, normalized by the time length of the embedding vector. We hypothesized that the IR regarded as a function of the embedding time delay (which is proportional to the length of the embedding vector) would be a good proxy to probe the dynamical system's time scales.

Although quantifying the complexity of activity patterns was not the goal of this study, we did measure PE, which is also based on the distribution of ordinal patterns. Our analysis suggests that OCD has a rather limited influence on the PE. Specifically, two small effects were observed (Figure 3): in two areas of the right hemisphere PE was somewhat larger in OCD patients than in controls over an interval of time scales that ranged from about one up to a few seconds. The other difference, which was observed in almost all channels, was found at short embedding times. Here, the PE saturated slower (i.e., at larger times) in OCD patients than in controls. This difference could be explained by the slower decay of the autocorrelation function. Overall, the PE does not seem a promising candidate as a discriminator of the two conditions. Since our aim was to investigate the extent to which the discriminatory power of the permutation-based irreversibility measure is redundant with respect to the IR, a comparison with other complexity measures goes beyond the scope of the present study.

The main part of our analysis was related to the behavior of the IR as a function of the embedding time delay τ, which made it possible to uncover significant differences in the brain activity of patients and controls. In this respect, we found that OCD does not seem to considerably alter the range in which the IR was significantly larger than the noise floor. This range corresponds to embedding delays in the interval 8 ms to 100 ms. Within this range, however, a clear difference was observed between OCD patients and the controls. In OCD patients, the IR was consistently concentrated in the “faster” (shorter time scales) part of the time-delay axis, whereas in HC the IR was larger at larger time delays or spread over the entire range, depending on the considered channel. To describe these two ranges of “fast” and “slow” time scales concisely, we introduced two intervals on the time-delay axis (see Figure 4). Notably, the IR in the “slow” range measured in OCD patients was larger than at baseline, so that the difference between patients and controls was milder in the second recording session following yogic therapy.

This major difference between the two groups was also evident when the average over all channels was considered (see Figure 5A), and when the embedding dimension was changed to *d* = 3 (see Figure 5B). Although the relative importance of fast and slow time scales persisted for *d* = 3, the IR measured at this smaller embedding dimension was generally lower (not shown). This fact gives a possible interpretation of why the IR rapidly drops to negligible values for all groups when the delay is increased beyond τ_3_. It is possible that this rapid decrease of the IR is due to an excessive coarse-graining, i.e., an insufficiently small embedding dimension cannot resolve higher IR over longer time windows. This methodological limitation is consistent with what was observed in other empirical estimates of irreversibility in living systems (54). In principle, using a higher embedding dimension could make it possible to resolve the IR for slower time scales. However, enlarging the embedding dimensions makes the size of the required data set increase exponentially. In spite of these practical limitations of the method, a clear distinction between the two groups was observed in the practically accessible range of time scales, and the mild increase of the IR in the slow range following therapy was observed also in the average over all channels.

To ascertain whether the difference in the fast and slow IR had a spatial structure, we defined the average IR within the two intervals: ω_*f*_ was defined as the fast IR, i.e., averaged within (τ_1_, τ_2_), and ω_*s*_ was defined as the slow IR, averaged within (τ_2_, τ_3_). The exact definition was given in Equation 9. The spatial distribution of both ω_*s*_ and ω_*f*_ revealed a clear difference between the two groups: in the HC group both fast and slow IR were larger in the channels located toward the back of both hemispheres, whereas ω_*s*_ and ω_*f*_ were rather uniform in OCD patients. In other words, the spatial distribution in control subjects was clearly non-uniform and asymmetric with respect to an axis roughly given by the intersection of the coronal plane with the two probes, as opposed to the essentially uniform and more symmetrical distribution seen in OCD patients (Figures 6–9). This asymmetry was quantified by the intrahemispheric asymmetry index β, defined in Equation 10. This asymmetry index was found to be clearly positive in HC in both hemispheres, but close to zero for OCD patients. Indeed, the spatial distribution of ω_*f*_ and ω_*s*_ was close to uniform in all cases. The difference between the two groups was particularly marked in the left hemisphere, for which the statistical model gave a statistically significant difference between the two groups, despite the rather limited number of subjects available for the analysis (see Figure 10). The yogic therapy did not seem to alter the asymmetry index appreciably. Although the average intrahemispheric asymmmetry of the slow IR increased, this effect was too small to be statistically significant given the limited number of available subjects.

Finally, we investigated whether OCD influenced the inter-hemispheric asymmetry. To this end, we computed the log-ratio between the fast or slow IR measured from homologous channels of the two hemispheres (Figures 11, 12). This part of our analysis suggested that OCD modulates this kind of asymmetry (Figure 13). In particular, the slow IR ω_*s*_ was, on the global level, predominant in the right hemisphere when control subjects were considered, whereas OCD patients displayed no clear hemispheric dominance in the slow part of the observed IR. The fast IR behaved differently: on the global level, it was rather balanced in HCs, whereas it was prevalent in the left hemisphere in OCD patients.

Concerning the interpretation of the IR, it could be tempting to invoke the fundamental result of stochastic thermodynamics that links the irreversibility of trajectories to a lower bound on the thermodynamic entropy production and, hence, to energy dissipation (125–129). However, this connection holds only when the transitions that make up the system's trajectories are energetically constrained, which is clearly not the case at hand. Even the interpretation as information entropy production must be cautiously applied here due to the peculiar nature of the coarse-graining induced by the conversion of the original time series into the ordinal pattern series. Even in very simple systems, different coarse-graining procedures can influence the information loss on the original system in different ways and lead to very different estimates on the entropy production (128, 130, 131). Despite these caveats, the quantification of irreversibility remains a useful way of characterizing the system's dynamical properties and the empirical evidence that time-reversal symmetry breaking is modulated by pathologies and even behavioral states corroborates this view (56–60).

These considerations substantiate the *a priori* expectation that many contingent variables could influence the IR, so that a change would not necessarily be an indication of pathology. Hence, the neurophysiological interpretation of the IR is not straightforward both for the theoretical reasons explained above and for the diversity of factors that can influence its magnitude. Indeed, in comparing the two recording sessions of the control group, a substantial difference in the absolute values of the IR was observed in most recording channels. Considering that the two recordings were taken 1 year apart, it is conceivable that many variables outside experimental control could cause this variability, which should hence not be regarded as surprising. Undoubtedly, the fact that recordings were performed only at two different time-points separated by a long period is a limitation of the study design, beside the moderate number of participants. These limitations notwithstanding, how the IR was distributed across time scales as well as the intra- and inter-hemispheric asymmetry in the spatial distributions were mostly consistent between the two recording sessions of control subjects, but in marked contrast to what was seen in OCD patients. Therefore, these asymmetries (or rather lack thereof) as well as the relative importance of slow and fast time scales quantified by the IR seem promising candidates to back up traditional analyses in the endeavor of pinpointing the elusive traces of obsessive compulsive disorder in neuroimaging data.

## Data availability statement

The raw data supporting the conclusions of this article will be made available by the authors, without undue reservation.

## Ethics statement

The study was conducted in compliance with the Code of Ethics of the World Medical Association, Declaration of Helsinki. The analysis of the MEG data continues to receive approval and coverage by the University of California, San Diego Human Subjects Internal Review Board. The patients/participants provided their written informed consent to participate in this study.

## Author contributions

DB and DP conceived the idea, designed the study, and wrote the first draft. DB performed all data processing and statistical analyses. DS-K, JS, and JW coordinated the data acquisition. DB, LF, and DP interpreted the results. All authors contributed to the final manuscript.

## References

[1] Kaplan AY Fingelkurts AA Fingelkurts AA Borisov SV Darkhovsky BS. Nonstationary nature of the brain activity as revealed by EEG/MEG: Methodological, practical and conceptual challenges. Signal Process. (2005) 85:2190–212. 10.1016/j.sigpro.2005.07.010

[2] Gilboa G Chen R Brenner N. History-dependent multiple-time-scale dynamics in a single-neuron model. J Neurosci. (2005) 25:6479–89. 10.1523/JNEUROSCI.0763-05.2005 16014709 PMC6725418

[3] Hasson U Yang E Vallines I Heeger DJ Rubin N. A hierarchy of temporal receptive windows in human cortex. J Neurosci. (2008) 28:2539–50. 10.1523/JNEUROSCI.5487-07.2008 18322098 PMC2556707

[4] Fingelkurts AA Fingelkurts AA Neves CFH. Natural world physical, brain operational, and mind phenomenal space–time. Phys Life Rev. (2010) 7:195–249. 10.1016/j.plrev.2010.04.001 20417160

[5] Papo D. Time scales in cognitive neuroscience. Front Physiol. (2013) 4:86. 10.3389/fphys.2013.00086 23626578 PMC3630296

[6] Chaudhuri R Bernacchia A Wang X.-J. A diversity of localized timescales in network activity. eLife. (2014) 3:e01239. 10.7554/eLife.01239 24448407 PMC3895880

[7] von Wegner F Tagliazucchi E Laufs H. Information-theoretical analysis of resting state EEG microstate sequences - non-markovianity, non-stationarity and periodicities. NeuroImage. (2017) 158:99–111. 10.1016/j.neuroimage.2017.06.062 28673879

[8] Papo D. How can we study reasoning in the brain? Front Hum Neurosci. (2015) 9:222. 10.3389/fnhum.2015.00222 25964755 PMC4408754

[9] Arieli A Sterkin A Grinvald A Aertsen A. Dynamics of ongoing activity: explanation of the large variability in evoked cortical responses. Science. (1996) 273:1868–71. 10.1126/science.273.5283.1868 8791593

[10] Deco G Jirsa VK McIntosh AR. Emerging concepts for the dynamical organization of resting-state activity in the brain. Nat Rev Neurosci. (2011) 12:43–56. 10.1038/nrn2961 21170073

[11] Kenet T Bibitchkov D Tsodyks M Grinvald A Arieli A. Spontaneously emerging cortical representations of visual attributes. Nature. (2003) 425:954–956. 10.1038/nature02078 14586468

[12] Beggs JM Plenz D. Neuronal avalanches in neocortical circuits. J Neurosci. (2003) 23:11167–77. 10.1523/JNEUROSCI.23-35-11167.2003 14657176 PMC6741045

[13] Ikegaya Y Aaron G Cossart R Aronov D Lampl I Ferster D. Synfire chains and cortical songs: temporal modules of cortical activity. Science. (2004) 304:559–64. 10.1126/science.1093173 15105494

[14] Dragoi G Tonegawa S. Preplay of future place cell sequences by hippocampal cellular assemblies. Nature. (2011) 469:397–401. 10.1038/nature09633 21179088 PMC3104398

[15] Novikov E Novikov A Shannahoff-Khalsa D Schwartz B Wright J. Scale-similar activity in the brain. Phys Rev E. (1997) 56:R2387–9. 10.1103/PhysRevE.56.R2387

[16] Linkenkaer-Hansen K Nikouline VV Palva JM Ilmoniemi RJ. Long-range temporal correlations and scaling behavior in human brain oscillations. J Neurosci. (2001) 21:1370–7. 10.1523/JNEUROSCI.21-04-01370.2001 11160408 PMC6762238

[17] Gong P Nikolaev AR van Leeuwen C. Scale-invariant fluctuations of the dynamical synchronization in human brain electrical activity. Neurosci Lett. (2003) 336:33–6. 10.1016/S0304-3940(02)01247-8 12493596

[18] Freeman WJ Holmes MD Burke BC Vanhatalo S. Spatial spectra of scalp EEG and EMG from awake humans. Clin Neurophysiol. (2003) 114:1053–68. 10.1016/S1388-2457(03)00045-2 12804674

[19] Allegrini P Menicucci D Paradisi P Gemignani A. Fractal complexity in spontaneous EEG metastable-state transitions: new vistas on integrated neural dynamics. Front Physiol. (2010) 1:128. 10.3389/fphys.2010.00128 21423370 PMC3059954

[20] Freyer F Aquino K Robinson PA Ritter P Breakspear M. Bistability and non-gaussian fluctuations in spontaneous cortical activity. J Neurosci. (2009) 29:8512–24. 10.1523/JNEUROSCI.0754-09.2009 19571142 PMC6665653

[21] Bianco S Ignaccolo M Rider M Ross M Winsor P Grigolini P. Brain, music, and non-poisson renewal processes. Phys Rev E. (2007) 75:61911. 10.1103/PhysRevE.75.061911 17677304

[22] Gong P Nikolaev AR van Leeuwen C. Intermittent dynamics underlying the intrinsic fluctuations of the collective synchronization patterns in electrocortical activity. Phys Rev E. (2007) 76:011904. 10.1103/PhysRevE.76.011904 17677491

[23] Palva J Zhigalov A Hirvonen J Korhonen O Linkenkaer-Hansen K Palva S. Neuronal long-range temporal correlations and avalanche dynamics are correlated with behavioral scaling laws. Proc Natl Acad Sci USA. (2013) 110:3585–90. 10.1073/pnas.1216855110 23401536 PMC3587255

[24] Linkenkaer-Hansen K Monto S Rytsälä H Suominen K Isometsä E Kähkänen S. Breakdown of long-range temporal correlations in theta oscillations in patients with major depressive disorder. J Neurosci. (2005) 25:10131–7. 10.1523/JNEUROSCI.3244-05.2005 16267220 PMC6725784

[25] Montez T Poil S.-S., Jones B, Manshanden I, Verbunt J, et al. Altered temporal correlations in parietal alpha and prefrontal theta oscillations in early-stage alzheimer disease. Proc Natl Acad Sci USA. (2009) 106:1614–9. 10.1073/pnas.0811699106 19164579 PMC2635782

[26] Linkenkaer-Hansen K Nikulin V Palva J Kaila K Ilmoniemi R. Stimulus-induced change in long-range temporal correlations and scaling behaviour of sensorimotor oscillations. Eur J Neurosci. (2004) 19:203–18. 10.1111/j.1460-9568.2004.03116.x 14750978

[27] Popivanov D Stomonyakov V Minchev Z Jivkova S Dojnov P Jivkov S. Multifractality of decomposed EEG during imaginary and real visual-motor tracking. Biol Cybern. (2006) 94:149–56. 10.1007/s00422-005-0037-5 16341722

[28] Buiatti M Papo D Baudonniére P-M Van Vreeswijk C. Feedback modulates the temporal scale-free dynamics of brain electrical activity in a hypothesis testing task. Neuroscience. (2007) 146:1400–12. 10.1016/j.neuroscience.2007.02.048 17418496

[29] He BJ Zempel JM Snyder AZ Raichle ME. The temporal structures and functional significance of scale-free brain activity. Neuron. (2010) 66:353–69. 10.1016/j.neuron.2010.04.020 20471349 PMC2878725

[30] Ciuciu P Abry P Rabrait C Wendt H. Log wavelet leaders cumulant based multifractal analysis of EVI fMRI time series: evidence of scaling in ongoing and evoked brain activity. IEEE J Sel Top Signal Process. (2008) 2:929–43. 10.1109/JSTSP.2008.2006663

[31] Zilber N Ciuciu P Abry P Van Wassenhove V. Modulation of Scale-Free Properties of Brain Activity in MEG. Barcelona: IEEE (2012).

[32] Suckling J Wink A Bernard F Barnes A Bullmore E. Endogenous multifractal brain dynamics are modulated by age, cholinergic blockade and cognitive performance. J Neurosci Meth. (2009) 174:292–300. 10.1016/j.jneumeth.2008.06.037 18703089 PMC2590659

[33] Papo D. Measuring brain temperature without a thermometer. Front Physiol. (2014) 5:124. 10.3389/fphys.2014.00124 24723893 PMC3973909

[34] Mackey M Glass L. Oscillation and chaos in physiological control systems. Science. (1977) 197:287–9. 10.1126/science.267326 267326

[35] Glass J Mackey M. Pathological conditions resulting from instabilities in physiological control systems. Ann NY Acad Sci. (1979) 316:214–35. 10.1111/j.1749-6632.1979.tb29471.x 288317

[36] Pezard L Nandrino JL Renault B El Massioui F Allilaire JF Müller J. Depression as a dynamical disease. Biol Psychiat. (1996) 39:991–9. 10.1016/0006-3223(95)00307-X 8780833

[37] Pezard L Martinerie J Varela F Bouchet F Guez D Derouesné C. Entropy maps characterize drug effects on brain dynamics in alzheimer's disease. Neurosci Lett. (1998) 253:5–8. 10.1016/S0304-3940(98)00603-X 9754791

[38] Glass L. Dynamical disease: challenges for nonlinear dynamics and medicine. Chaos. (2015) 25:097603. 10.1063/1.4915529 26428556

[39] Abarbanel H Brown R Sidorowich J Tsimring L. The analysis of observed chaotic data in physical systems. Rev Modern Phys. (1993) 65:1331–92. 10.1103/RevModPhys.65.1331

[40] Rezek I Roberts S. Stochastic complexity measures for physiological signal analysis. IEEE Trans Biomed Eng. (1998) 45:1186–91. 10.1109/10.709563 9735569

[41] Hegger R Kantz H Schreiber T. Practical implementation of nonlinear time series methods: the TISEAN package. Chaos. (1999) 9:413–35. 10.1063/1.166424 12779839

[42] Kantz H Schreiber T. Nonlinear Time Series Analysis, Vol. 7 Cambridge: Cambridge University Press (2004).

[43] Molnár M. Brain complexity as revealed by non-linear and linear electrophysiology. Int J Psychophysiol. (1999) 34:1–3. 10.1016/S0167-8760(99)00037-9 10555869

[44] Pezard L Jech R Rǔžička E. Investigation of non-linear properties of multichannel EEG in the early stages of Parkinson's disease. Clin Neurophysiol. (2001) 112:38–45. 10.1016/S1388-2457(00)00512-5 11137659

[45] Lehnertz K Andrzejak R Arnhold J Kreuz T Mormann F Rieke C. Nonlinear EEG analysis in epilepsy: its possible use for interictal focus localization, seizure anticipation, and prevention. J Clin Neurophysiol. (2001) 18:209–22. 10.1097/00004691-200105000-00002 11528294

[46] Stam C. Nonlinear dynamical analysis of EEG and MEG: Review of an emerging field. Clin Neurophysiol. (2005) 116:2266–301. 10.1016/j.clinph.2005.06.011 16115797

[47] Gnesotto F Mura F Gladrow J Broedersz C. Broken detailed balance and non-equilibrium dynamics in living systems: a review. Rep Progr Phys. (2018) 81:066601. 10.1088/1361-6633/aab3ed 29504517

[48] Pomeau Y. Symétrie des fluctuations dans le renversement du temps. J Phys. (1982) 43:859–67. 10.1051/jphys:01982004306085900

[49] Weiss G. Time-reversibility of linear stochastic processes. J Appl Probab. (1975) 12:831–6. 10.1017/S002190020004880415244905

[50] Lawrance A. Directionality and reversibility in time series. Int Stat Rev. (1991) 59:67. 10.2307/1403575

[51] Stone L Landan G May R. Detecting time's arrow: a method for identifying nonlinearity and deterministic chaos in time-series data. Proc R Soc Lond B Biol Sci. (1996) 263:1509–13. 10.1098/rspb.1996.0220

[52] Gallavotti G. Fluctuation relation, fluctuation theorem, thermostats and entropy creation in nonequilibrium statistical physics. C R Phys. (2007) 8:486–94. 10.1016/j.crhy.2007.04.011

[53] Egolf D. Equilibrium regained: from nonequilibrium chaos to statistical mechanics. Science. (2000) 287:101–4. 10.1126/science.287.5450.101 10615038

[54] Tan TH Watson GA Chao Y.-C., Li J, Gingrich TR, et al. Scale-dependent irreversibility in living matter. arXiv. (2021).

[55] Paluš M. Nonlinearity in normal human EEG: cycles, temporal asymmetry, nonstationarity and randomness, not chaos. Biol Cyber. (1996) 75:389–96. 10.1007/s004220050304 8983161

[56] Zanin M Güntekin B Aktürk T Hanoğlu L Papo D. Time irreversibility of resting-state activity in the healthy brain and pathology. Front Physiol. (2020) 10:1619. 10.3389/fphys.2019.01619 32038297 PMC6987076

[57] Deco G Perl YS Sitt JD Tagliazucchi E Kringelbach ML. Deep learning the arrow of time in brain activity: characterising brain-environment behavioural interactions in health and disease. bioRxiv. (2021) 10.1101/2021.07.02.450899

[58] De La Fuente L Zamberlan F Bocaccio H Kringelbach M Deco G Sanz Perl Y. Temporal irreversibility of neural dynamics as a signature of consciousness. Cereb Cortex. (2022) 177:802. 10.1101/2021.09.02.458802 35512291

[59] Schindler K Rummel C Andrzejak R Goodfellow M Zubler F Abela E. Ictal time-irreversible intracranial EEG signals as markers of the epileptogenic zone. Clin Neurophysiol. (2016) 127:3051–8. 10.1016/j.clinph.2016.07.001 27472540

[60] Martínez JH Herrera-Diestra JL Chavez M. Detection of time reversibility in time series by ordinal patterns analysis. Chaos. (2018) 28:123111. 10.1063/1.5055855 30599517

[61] American Psychiatric Association. Diagnostic and Statistical Manual of Mental Disorders. 5th ed. Washington, DC: American Psychiatric Publishing (2013).

[62] Koran L Hanna G Hollander E Nestadt G Simpson H. Practice guideline for the treatment of patients with obsessive-compulsive disorder. Am J Psychiatry. (2006) 164:5–53. 10.4088/jcp.v67n0411 17849776

[63] Pittenger C. editors. Obsessive-Compulsive Disorder: Phenomenology, Pathophysiology, Treatment. New York, NY: Oxford University Press (2017).

[64] Christensen H Hadzi-Pavlovic D Andrews G Mattick R. Behavior therapy and tricyclic medication in the treatment of obsessive-compulsive disorder: a quantitative review. J Consult Clin Psychol. (1987) 55:701–11. 10.1037/0022-006X.55.5.701 3331632

[65] Abramowitz JS. Effectiveness of psychological and pharmacological treatments for obsessive-compulsive disorder: a quantitative review. J Consult Clin Psychol. (1997) 65:44–52. 10.1037/0022-006X.65.1.44 9103733

[66] Soomro G Altman D Rajagopal S Oakley Browne M. Selective serotonin re-uptake inhibitors (ssris) versus placebo for obsessive compulsive disorder (OCD). Cochrane Db Syst Rev. (2008) 2008:D001765. 10.1002/14651858.CD001765.pub3 18253995 PMC7025764

[67] Fineberg NA Reghunandanan S Simpson HB Phillips KA Richter MA Matthews K. Obsessive–compulsive disorder (OCD): practical strategies for pharmacological and somatic treatment in adults. Psychiat Res. (2015) 227:114–25. 10.1016/j.psychres.2014.12.003 25681005

[68] Craske M Treanor M Conway C Zbozinek T Vervliet B. Maximizing exposure therapy: an inhibitory learning approach. Behav Res Ther. (2014) 58:10–23. 10.1016/j.brat.2014.04.006 24864005 PMC4114726

[69] Öst LG Havnen A Hansen B Kvale G. Cognitive behavioral treatments of obsessive–compulsive disorder. A systematic review and meta-analysis of studies published 1993–2014. Clin Psychol Rev. (2015) 40:156–69. 10.1016/j.cpr.2015.06.003 26117062

[70] Tastevin M Spatola G Régis J Lançon Lançon C Richieri R. Deep brain stimulation in the treatment of obsessive-compulsive disorder: current perspectives. Neuropsych Dis Treat. (2019) 15:1259–72. 10.2147/NDT.S178207 31190832 PMC6526924

[71] Balzus L Klawohn J Elsner B Schmidt S Brandt S Kathmann N. Non-invasive brain stimulation modulates neural correlates of performance monitoring in patients with obsessive-compulsive disorder. Neuroimage Clin. (2022) 35:103113. 10.1016/j.nicl.2022.103113 35870380 PMC9421486

[72] Shannahoff-Khalsa DS Beckett LR. Clinical case report: Efficacy of yogic techniques in the treatment of obsessive compulsive disorders. Int J Neurosci. (1996) 85:1–17. 10.3109/00207459608986347 8727678

[73] Shannahoff-Khalsa D. Yogic Techniques Are Effective in the Treatment of Obsessive Compulsive Disorders. New York, NY: Marcel Dekker Inc. (1997).

[74] Shannahoff-Khalsa DS Ray LE Levine S Gallen CC Schwartz BJ Sidorowich JJ. Randomized controlled trial of yogic meditation techniques for patients with obsessive-compulsive disorder. CNS Spectrums. (1999) 4:34–47. 10.1017/S1092852900006805 18311106

[75] Shannahoff-Khalsa DS. Kundalini yoga meditation techniques in the treatment of obsessive compulsive and OC spectrum disorders. Brief Treat Crisis Intervention. (2003) 3:369–82. 10.1093/brief-treatment/mhg027

[76] Shannahoff-Khalsa D. Kundalini Yoga meditation techniques in the treatment of obsessive compulsive and OC spectrum disorders. In: *Chapter 86 in*: Albert Roberts R, editor. Social Workers' Desk Reference. 2nd ed, New York, NY: Oxford University Press (2008). p. 606–12.

[77] Shannahoff-Khalsa D Fernandes RY Pereira CAdB March JS Leckman JF. Kundalini yoga meditation versus the relaxation response meditation for treating adults with obsessive-compulsive disorder: A randomized clinical trial. Front Psychiatry. (2019) 10:793. 10.3389/fpsyt.2019.00793 31780963 PMC6859828

[78] Cavedini P Ferri S Scarone S Bellodi L. Frontal lobe dysfunction in obsessive-compulsive disorder and major depression: a clinical-neuropsychological study. Psychiat Res. (1998) 78:21–8. 10.1016/S0165-1781(97)00153-4 9579699

[79] Cavallaro R Cavedini P Mistretta P Bassi T Angelone S Ubbiali A. Basal-corticofrontal circuits in schizophrenia and obsessive-compulsive disorder. Biol Psychiat. (2003) 54:437–43. 10.1016/S0006-3223(02)01814-0 12915288

[80] Menzies L Chamberlain S Laird A Thelen S Sahakian B Bullmore E. Integrating evidence from neuroimaging and neuropsychological studies of obsessive-compulsive disorder: The orbitofronto-striatal model revisited. Neurosci Biobehav Rev. (2008) 32:525–49. 10.1016/j.neubiorev.2007.09.005 18061263 PMC2889493

[81] Beucke J Sepulcre J Talukdar T Linnman C Zschenderlein K Endrass T. Abnormally high degree connectivity of the orbitofrontal cortex in obsessive-compulsive disorder. JAMA Psychiatry. (2013) 70:619. 10.1001/jamapsychiatry.2013.173 23740050

[82] Brennan B Rauch S. Functional Neuroimaging Studies in Obsessive-Compulsive Disorder: Overview and Synthesis. New York, NY: Oxford University Press (2017). p. 213–30.

[83] Khanna S. Obsessive-compulsive disorder: is there a frontal lobe dysfunction? Biol Psychiat. (1988) 24:602–13. 10.1016/0006-3223(88)90171-0 3048425

[84] Perera M Bailey N Herring S Fitzgerald P. Electrophysiology of obsessive compulsive disorder: a systematic review of the electroencephalographic literature. J Anxiety Disord. (2019) 62, 1-14. 10.1016/j.janxdis.2018.11.001 30469123

[85] Zaboski B Stern E Skosnik P Pittenger C. Electroencephalographic correlates and predictors of treatment outcome in OCD: a brief narrative review. Front Psychiatry. (2021) 12:703398. 10.3389/fpsyt.2021.703398 34408681 PMC8365146

[86] Morault P Bourgeois M Laville J Bensch C Paty J. Psychophysiological and clinical value of event-related potentials in obsessive-compulsive disorder. Biol Psychiat. (1997) 42:46–56. 10.1016/S0006-3223(96)00228-4 9193741

[87] Andreou C Leicht G Popescu V Pogarell O Mavrogiorgou P Rujescu D. P300 in obsessive–compulsive disorder: source localization and the effects of treatment. J Psychiat Res. (2013) 47:1975–83. 10.1016/j.jpsychires.2013.09.003 24075207

[88] Jansen M De Bruijn E. Mistakes that matter: An event-related potential study on obsessive-compulsive symptoms and social performance monitoring in different responsibility contexts. Cogn Affect Behav Neurosci. (2020) 20:684–97. 10.3758/s13415-020-00796-3 32372323 PMC7394925

[89] Molina V Montz R Pérez-Castejón M Martín-Loeches M Carreras J Calcedo A. Cerebral perfusion, electrical activity and effects of serotonergic treatment in obsessive-compulsive disorder. Neuropsychobiology. (1995) 32:139–48. 10.1159/000119227 8544971

[90] Tot Ş Özge A Çömelekoğlu U Yazici K Bal N. Association of QEEG findings with clinical characteristics of OCD: evidence of left frontotemporal dysfunction. Can J Psychiatry. (2002) 47:538–45. 10.1177/070674370204700605 12211881

[91] Karadag F Oguzhanoglu N Kurt T Oguzhanoglu A Atesci F Özdel O. Quantitative EEG analysis in obsessive compulsive disorder. Int J Neurosci. (2003) 113:833–47. 10.1080/00207450390200963 12775347

[92] Bucci P Mucci A Volpe U Merlotti E Galderisi S Maj M. Executive hypercontrol in obsessive–compulsive disorder: electrophysiological and neuropsychological indices. Clin Neurophysiol. (2004) 115:1340–8. 10.1016/j.clinph.2003.12.031 15134701

[93] Pogarell O Juckel G Mavrogiorgou P Mulert C Folkerts M Hauke W. Symptom-specific EEG power correlations in patients with obsessive–compulsive disorder. Int J Psychophysiol. (2006) 62:87–92. 10.1016/j.ijpsycho.2006.02.002 16554100

[94] Velikova S Locatelli M Insacco C Smeraldi E Comi G Leocani L. Dysfunctional brain circuitry in obsessive–compulsive disorder: source and coherence analysis of EEG rhythms. NeuroImage. (2010) 49:977–83. 10.1016/j.neuroimage.2009.08.015 19683062

[95] Kopřivová J Congedo M Horáček J Praško J Raszka M Brunovský M. EEG source analysis in obsessive–compulsive disorder. Clin Neurophysiol. (2011) 122:1735–43. 10.1016/j.clinph.2011.01.051 21354363

[96] Kopřivová J Horáček J Raszka M Brunovský M Praško J. Standardized low-resolution electromagnetic tomography in obsessive–compulsive disorder–a replication study. Neurosci Lett. (2013) 548:185–9. 10.1016/j.neulet.2013.05.015 23701862

[97] Olbrich S Olbrich H Adamaszek M Jahn I Hegerl U Stengler K. Altered EEG lagged coherence during rest in obsessive–compulsive disorder. Clin Neurophysiol. (2013) 124:2421–30. 10.1016/j.clinph.2013.05.031 23968842

[98] Kamaradova D Prasko J Taborsky J Grambal A Latalova K Hajda M. Cognitive deficits in patients with obsessive-compulsive disorder - electroencephalography correlates. Neuropsych Dis Treat. (2016) 1119:3040. 10.2147/NDT.S93040 27226716 PMC4866747

[99] Newson J Thiagarajan T. EEG frequency bands in psychiatric disorders: a review of resting state studies. Front Hum Neurosci. (2019) 12:521. 10.3389/fnhum.2018.00521 30687041 PMC6333694

[100] Kuskowski M Malone S Kim S Dysken M Okaya A Christensen K. Quantitative EEG in obsessive-compulsive disorder. Biol Psychiat. (1993) 33:423–30. 10.1016/0006-3223(93)90170-I 8490069

[101] Desarkar P Das A Nizamie S. Aripiprazole-induced obsessive-compulsive disorder. J Clin Psychopharm. (2007) 27:305–6. 10.1097/01.jcp.0000270091.32286.0a 17502782

[102] Wong M Woody E Schmidt L Ameringen M Soreni N Szechtman H. Frontal EEG alpha activity and obsessive-compulsive behaviors in non-clinical young adults: a pilot study. Front Psychol. (2015) 6:1480. 10.3389/fpsyg.2015.01480 26483733 PMC4586322

[103] Ischebeck M Endrass T Simon D Kathmann N. Altered frontal EEG asymmetry in obsessive-compulsive disorder. Psychophysiology. (2014) 51:596–601. 10.1111/psyp.12214 24673721

[104] Özçoban M Tan O Aydin S Akan A. Decreased global field synchronization of multichannel frontal EEG measurements in obsessive-compulsive disorders. Med Biol Eng Comput. (2018) 56:331–8. 10.1007/s11517-017-1689-8 28741170

[105] Ozel P Karaca A Olamat A Akan A Ozcoban M Tan O. Intrinsic synchronization analysis of brain activity in obsessive–compulsive disorders. Int J Neural Syst. (2020) 30:2050046. 10.1142/S012906572050046X 32902344

[106] Tan B Liu Q Wan C Jin Z Yang Y Li L. Altered functional connectivity of alpha rhythm in obsessive-compulsive disorder during rest. Clin EEG Neurosci. (2019) 50:88–99. 10.1177/1550059418804378 30280595

[107] Choi KM Kim JY Kim YW Han JW Im CH Lee SH. Comparative analysis of default mode networks in major psychiatric disorders using resting-state EEG. Sci Rep. (2021) 11:22007. 10.1038/s41598-021-03736-4 34759276 PMC8580995

[108] Baglio S Bucolo M Fortuna L Frasca M La Rosa M Shannahoff-Khalsa D. MEG signals spatial power distribution and gamma band activity in yoga breathing exercises. In: Proceedings of the Second Joint 24th Annual Conference and the Annual Fall Meeting of the Biomedical Engineering Society] [*Engineering in Medicine and Biology, Vol.1*. Houston, TX: IEEE (2002). p. 175–76.

[109] Bucolo M Di Grazia F Frasca M Sapuppo F Shannahoff-Khalsa D. From synchronization to network theory: a strategy for MEG data analysis. in 2008 16th Mediterranean Conference on Control and Automation. Ajaccio: IEEE (2008). p. 854–9.27534393

[110] Aydin S Arica N Ergul E Tan O. Classification of obsessive compulsive disorder by EEG complexity and hemispheric dependency measurements. Int J Neural Syst. (2015) 25:1550010. 10.1142/S0129065715500100 25804351

[111] Tan O Aydin S. Electroencephalographic complexity and decreased randomness in drug-naive obsessive-compulsive patients. Dusunen Adam. (2017) 30:101–12. 10.5350/DAJPN2017300204

[112] Yazdi-Ravandi S Shamsaei F Matinnia N Moghimbeigi A Shams J Ahmadpanah M. Executive functions, selective attention and information processing in patients with obsessive compulsive disorder: a study from west of iran. Asian J Psychiatr. (2021) 37:140–5. 10.1016/j.ajp.2018.09.002 30223238

[113] Altuğlu T Metin B Tülay E Tan O Sayar G Taş C. Prediction of treatment resistance in obsessive compulsive disorder patients based on EEG complexity as a biomarker. Clin Neurophysiol. (2020) 131:716–24. 10.1016/j.clinph.2019.11.063 32000072

[114] Zanin M Papo D. Algorithmic approaches for assessing irreversibility in time series: Review and comparison. Entropy. (2021) 23:1474. 10.3390/e23111474 34828172 PMC8622570

[115] Graff G Graff B Kaczkowska A Makowiec D Amigó JM Piskorski J. Ordinal pattern statistics for the assessment of heart rate variability. Eur Phys J Special Topics. (2013) 222:525–34. 10.1140/epjst/e2013-01857-428527351

[116] Zanin M Rodríguez-González A Menasalvas Ruiz E Papo D. Assessing time series reversibility through permutation patterns. Entropy. (2018) 20:665. 10.3390/e20090665 33265754 PMC7513188

[117] Martínez JH Ramasco JJ Zanin M. On the complementarity of ordinal patterns-based entropy and time asymmetry metrics. Chaos. (2023) 33:033138. 10.1063/5.0136471 37003799

[118] Bandt C Pompe B. Permutation entropy: a natural complexity measure for time series. Phys Rev Lett. (2002) 88:174102. 10.1103/PhysRevLett.88.174102 12005759

[119] Bandt C. Ordinal time series analysis. Ecol Model. (2005) 182:229–38. 10.1016/j.ecolmodel.2004.04.003

[120] Zanin M Zunino L Rosso OA Papo D. Permutation entropy and its main biomedical and econophysics applications: a review. Entropy. (2012) 14:1553–77. 10.3390/e14081553

[121] Riedl M Müller A Wessel N. Practical considerations of permutation entropy. Eur Phys J Special Topics. (2013) 222:249–62. 10.1140/epjst/e2013-01862-7

[122] Yao W Yao W Wang J Dai J. Quantifying time irreversibility using probabilistic differences between symmetric permutations. Phys Lett A. (2019) 383:738–43. 10.1016/j.physleta.2018.11.043

[123] Bernardi D Lindner B. A frequency-resolved mutual information rate and its application to neural systems. J Neurophysiol. (2015) 113:1342–57. 10.1152/jn.00354.2014 25475346

[124] Schreiber T Schmitz A. Improved surrogate data for nonlinearity tests. Phys Rev Lett. (1996) 77:635–8. 10.1103/PhysRevLett.77.635 10062864

[125] Maes C Netočný K. Time-reversal and entropy. J Stat Phys. (2003) 110:269–310. 10.1023/A:1021026930129

[126] Gaspard P. Brownian motion, dynamical randomness and irreversibility. New J Phys. (2005) 7:77. 10.1088/1367-2630/7/1/077

[127] Kawai R Parrondo JMR Van den Broeck C. Dissipation: The phase-space perspective. Phys Rev Lett. (2007) 98:080602. 10.1103/PhysRevLett.98.080602 17359081

[128] Seifert U. From stochastic thermodynamics to thermodynamic inference. Annu Rev Condens Matter Phys. (2019) 10:171–92. 10.1146/annurev-conmatphys-031218-013554

[129] Peliti L Pigolotti S. Stochastic Thermodynamics. Princeton, NJ: Princeton University Press (2021).

[130] Gomez-Marin A Parrondo JMR Van den Broeck C. Lower bounds on dissipation upon coarse graining. Phys Rev E. (2008) 78:011107. 10.1103/PhysRevE.78.011107 18763919

[131] Esposito M. Stochastic thermodynamics under coarse graining. Phys Rev E. (2012) 85:041125. 10.1103/PhysRevE.85.041125 22680437

